# The *Vibrio cholerae* SpeG Spermidine/Spermine *N*-Acetyltransferase Allosteric Loop and β6-β7 Structural Elements Are Critical for Kinetic Activity

**DOI:** 10.3389/fmolb.2021.645768

**Published:** 2021-04-13

**Authors:** Van Thi Bich Le, Sofiya Tsimbalyuk, Ee Qi Lim, Allan Solis, Darwin Gawat, Paloma Boeck, Ee Qing Lim, Rosselini Renolo, Jade K. Forwood, Misty L. Kuhn

**Affiliations:** ^1^ Department of Chemistry & Biochemistry, San Francisco State University, San Francisco, CA, United States; ^2^ School of Biomedical Science, Charles Sturt University, Wagga Wagga, NSW, Australia

**Keywords:** Gcn5-related N-acetyltransferase, spermidine/spermine N-acetyltransferase, polyamine, allosteric regulation, acetylation, spermidine, spermine, chimeric

## Abstract

Polyamines regulate many important biological processes including gene expression, intracellular signaling, and biofilm formation. Their intracellular concentrations are tightly regulated by polyamine transport systems and biosynthetic and catabolic pathways. Spermidine/spermine *N*-acetyltransferases (SSATs) are catabolic enzymes that acetylate polyamines and are critical for maintaining intracellular polyamine homeostasis. These enzymes belong to the Gcn5-related *N*-acetyltransferase (GNAT) superfamily and adopt a highly conserved fold found across all kingdoms of life. SpeG is an SSAT protein found in a variety of bacteria, including the human pathogen *Vibrio cholerae.* This protein adopts a dodecameric structure and contains an allosteric site, making it unique compared to other SSATs. Currently, we have a limited understanding of the critical structural components of this protein that are required for its allosteric behavior. Therefore, we explored the importance of two key regions of the SpeG protein on its kinetic activity. To achieve this, we created various constructs of the *V. cholerae* SpeG protein, including point mutations, a deletion, and chimeras with residues from the structurally distinct and non-allosteric human SSAT protein. We measured enzyme kinetic activity toward spermine for ten constructs and crystallized six of them. Ultimately, we identified specific portions of the allosteric loop and the β6-β7 structural elements that were critical for enzyme kinetic activity. These results provide a framework for further study of the structure/function relationship of SpeG enzymes from other organisms and clues toward the structural evolution of members of the GNAT family across domains of life.

## Introduction

Polyamines are organic molecules that are fully protonated at physiological pH and bind to negatively charged sites of DNA, RNA, proteins, and phospholipid membranes (Tabor and Tabor, 1984; Finger et al., 2014). Examples of polyamines include putrescine (put), cadaverine (cad), spermidine (spd), spermine (spm), and norspermidine (nspd), but a variety of other polyamines exist (Hamana and Matsuzaki, 1992; Michael, 2016). They play many important roles in cellular processes, including the regulation of gene expression (Childs et al., 2003), intracellular signaling (Karatan et al., 2005; Sobe et al., 2017), virulence (Jelsbak et al., 2012), and biofilm formation (Karatan et al., 2005; Nesse et al., 2015; Sobe et al., 2017). Furthermore, polyamines are present in all domains of life and some viruses (Michael, 2016; Firpo and Mounce, 2020); however, some organisms within these groups do not contain any polyamines (Hamana and Matsuzaki, 1992; Pegg and Casero, 2011). The most common polyamines in bacteria are spd and put, but others such as nspd and spm can also be present (Hamana and Matsuzaki, 1992; Michael, 2016). In general, spd is found in high concentrations in bacteria, but some bacteria, including *Escherichia coli,* have a higher prevalence of put than spd. While most bacteria contain both of these polyamines, some bacteria either do not have spd or put, or they have different polyamines. For example, Vibrionaceae family members, including *Vibrio*, *Photobacterium*, and *Listonella* species, have nspd as the predominant polyamine (Hamana and Matsuzaki, 1992).

The concentrations of polyamines in bacteria are typically in the millimolar range (from 0.1 to 30 mM) (Shah and Swiatlo, 2008) and are tightly regulated by a variety of means, including polyamine import/export systems, and intracellular anabolic and catabolic processes (Casero and Pegg, 1993; Wallace et al., 2003; Pegg and Casero, 2011; Schneider and Wendisch, 2011). Catabolic enzymes that are critical for regulating polyamine concentrations include the spermidine/spermine *N*-acetyltransferases (SSATs). These enzymes acetylate polyamines using acetyl coenzyme A (AcCoA) as an acetyl donor (Zhu et al., 2006; Hegde et al., 2007; Filippova et al., 2015a; Tsimbalyuk et al., 2020). Once acetylated, the overall charge of the polyamine is reduced and the molecules are exported (Kramer et al., 2008) or recycled (Floris and Finazzi Agrò, 2013). Several different SSATs have been investigated, including hSSAT1 (*Homo sapiens*) (Zhu et al., 2006), PaiA (*Thermoplasma acidophilum, Bacillus subtillis*) (Forouhar et al., 2005; Filippova et al., 2011), BltD (*Bacillus subtilis*) (Woolridge et al., 1999), and SpeG (*Vibrio cholerae, E. coli, Coxiella burnetti, Staphylococcus aureus,* and *Bacillus thuringiensis*) (Fukuchi et al., 1994; Filippova et al., 2015a; Franklin et al., 2015; Li et al., 2019; Tsimbalyuk et al., 2020). All SSATs that have been kinetically characterized have been shown to acetylate spm more readily or at least as well as other polyamines. For quite some time it was thought that spm was not produced by bacteria and was instead imported into bacterial cells because they lack a spermine synthase (Ikeguchi et al., 2006). However, it has been shown that spm can indeed be produced in some bacteria, including some species of Actinobacteria, Clostridiales, and Bacilliales (Pegg and Michael, 2010; Michael, 2016), using a different mechanism that involves a carboxyspermidine dehydrogenase and carboxyspermidine decarboxylase (Kim et al., 2016).

While SSAT enzymes catalyze the same reaction and exhibit relatively similar substrate specificity, their 3D structures can differ quite significantly, but all retain a core Gcn5-related *N-*acetyltransferase (GNAT) structural fold. SpeG from *Vibrio cholerae* (VcSpeG) was the first GNAT enzyme shown to exhibit a dodecameric structure, which is unusual for the GNAT family of enzymes since they are predominantly monomers or lower ordered oligomers. VcSpeG is also the first SSAT enzyme to exhibit an allosteric site where polyamines can bind (Filippova et al., 2015a). The allosteric site is located between monomers that form the hexamers of the dodecamer (Filippova et al., 2015a), and some evidence has been provided that shows when polyamine binds to these sites, the dodecamer tightens (Filippova et al., 2015b). When apo- and polyamine-bound structures of VcSpeG are compared, there is a large conformational change of the allosteric loop that occurs (Filippova et al., 2015a).

**FIGURE 1:**
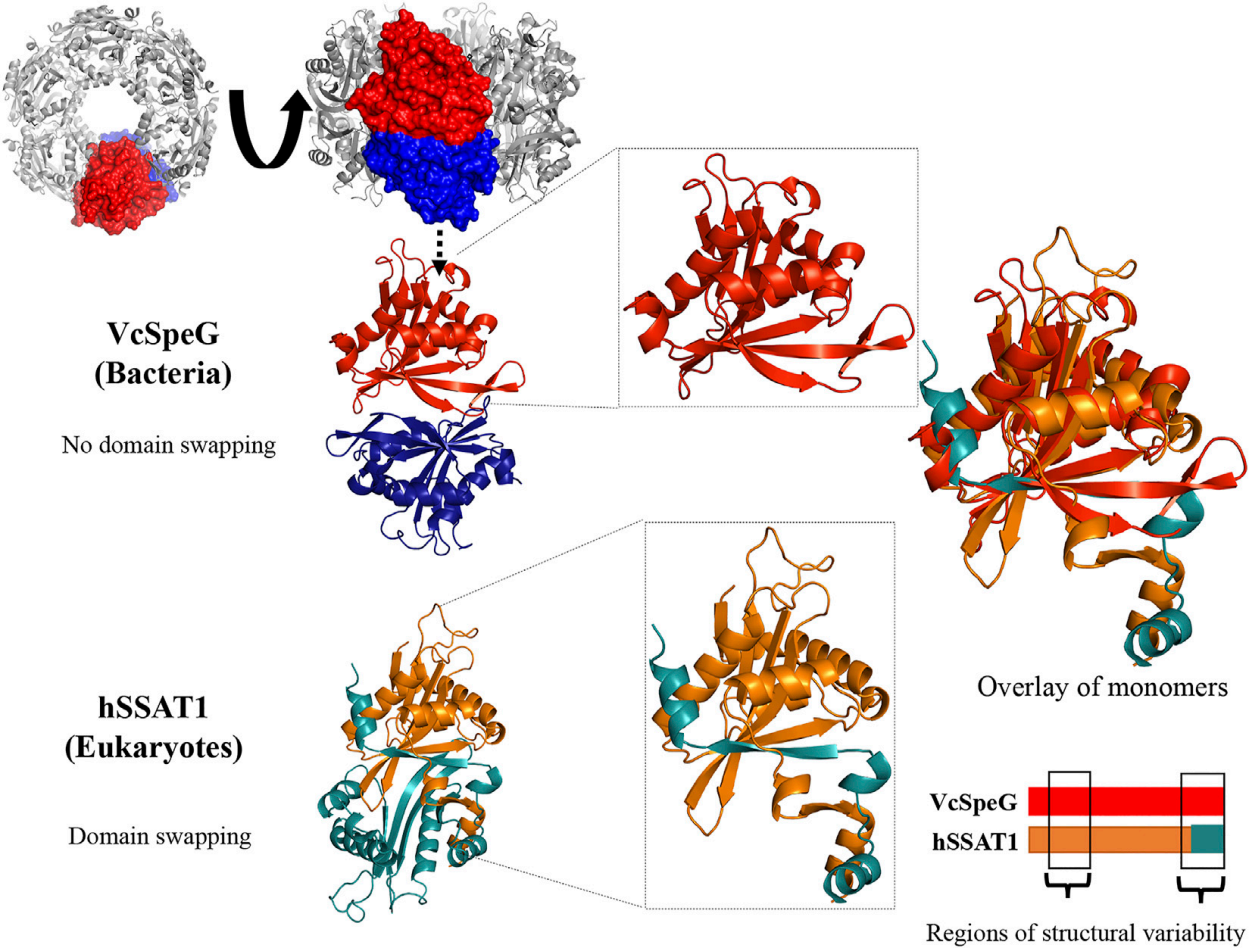
VcSpeG dodecamer and hSSAT dimer. The ribbon diagram and surface view for the VcSpeG (PDB ID: 4r87) dodecamer, dimer, and monomer are shown in gray, red and blue. The dodecamer is comprised of two hexamers on top of each other. Two monomers of different hexamers of the dodecamer are in red and blue and a zoomed view shows a single monomer. No domain swapping occurs between these monomers. The ribbon diagram of the hSSAT1 protein (PDB ID: 2b4b) monomers of the dimer are shown in orange and teal. Domain swapping occurs between two monomers, and a zoomed view of the swapped region is shown. An overlay of both monomers of the VcSpeG and hSSAT1 protein structures and regions of variability between the two proteins is shown.

To learn more about the importance of the allosteric loop and other regions of VcSpeG on its kinetic activity, we designed point mutants and a series of chimeric constructs between the structurally divergent hSSAT1 and VcSpeG proteins. These specific enzymes were selected because they were the most extreme cases of structural diversity of SSATs that have been characterized (Figure 1). Moreover, the importance of the allosteric loop of SpeG proteins has not been kinetically elucidated. Here, we identified specific residues of the allosteric loop and the β6-β7 region of the VcSpeG protein that are required for its enzymatic activity, which provides new insight into the structure/function relationship of SSAT proteins.

## Materials and Methods

### Materials

All chemicals were purchased at the highest quality from either Millipore Sigma or Thermo Fisher Scientific. Spermine dihydrate and acetyl coenzyme A trilithium salts were purchased from Millipore Sigma.

### Cloning

**FIGURE 2:**
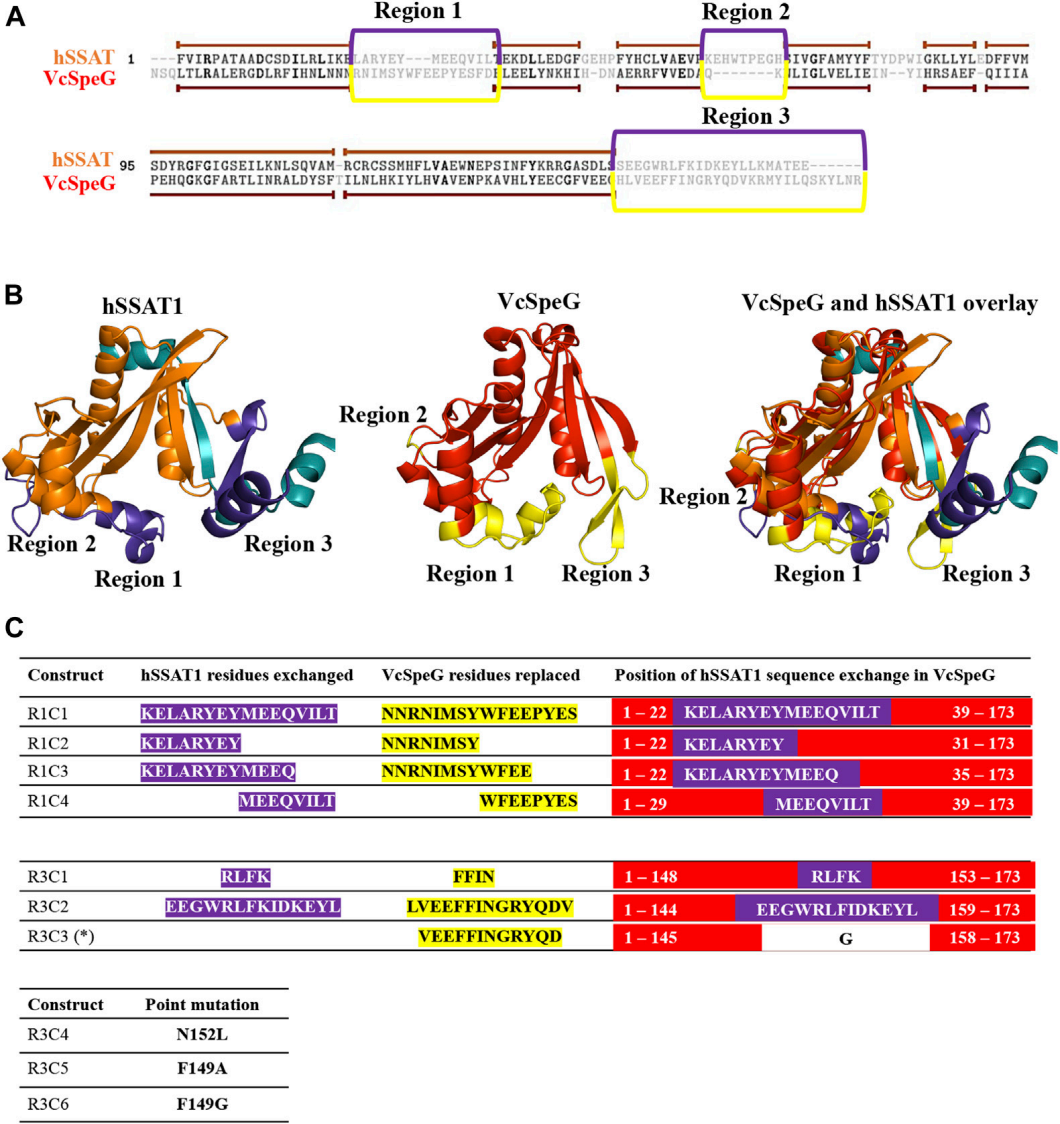
Comparison of sequences and structures of VcSpeG and hSSAT1 and defining regions and constructs for this study. **A**. Dissimilar regions between VcSpeG (PDB ID: 4r87) and hSSAT1 (PDB ID: 2b4b) structures shown on a primary sequence alignment generated using TopMatch. Gray residues framed in purple (hSSAT1) and yellow (VcSpeG) show unaligned regions of the structures during structural alignment. We named these regions of structural variability in sequential order. **B**. Three different regions highlighted on the monomer VcSpeG and hSSAT1 structures. All three regions are shown in purple on the hSSAT1 structure (orange); the teal secondary structure shown is from the adjacent monomer of the dimer. Only the complete monomer is shown for clarity. Similarly, all three regions of the VcSpeG protein monomer (red) are highlighted in yellow. **C.** Construct names and residues substituted or altered in the ten VcSpeG constructs for this study. Residues highlighted in yellow are removed from VcSpeG and replaced by hSSAT1 residues in purple. The position of hSSAT1 exchange on each VcSpeG cartoon linear protein sequence is also shown. An asterisk indicates the deletion construct.

Different constructs (outlined in Figure 2, Supplementary Figure S1 and Table S1) were made using the Q5® site-directed mutagenesis kit (New England Biolabs, Inc.) and the VcSpeG plasmid template (*V. cholerae O1 biovar El Tor str. N16961 speG* (VC_A0947) gene*,* Uniprot ID: Q9KL03) described in (Majorek et al., 2014; Filippova et al., 2015a). Forward and reverse primers (Supplementary Table S1) were designed using the NEBasechanger tool (https://nebasechanger.neb.com/) and purchased from Integrated DNA Technologies, Inc. A 50 µL Polymerase Chain Reaction (PCR) (1 ng VcSpeG template DNA; 0.25 µM forward and reverse primers; and 1X master mix) was performed using an Axygen® MaxyGene II Thermal Cycler. Denaturation, annealing and extension times and temperatures were selected based on the manufacturer’s recommendations and the NEBasechanger tool output for each set of primers for individual constructs. The size of PCR products was confirmed on a 1% agarose gel and then samples were subjected to the kinase, ligase, and DpnI reaction (1 µL PCR product, 1X KLD buffer, and 1X KLD enzyme in a final volume of 10 µL) for 5 min at RT. Then 0.5 µL of this product was used to transform 100 µL of DH5α competent cells that were previously prepared using the Zymo Research Mix & Go transformation kit. We spread 20 µL of the transformation mixture and 20 µL of SOC media onto an LB-agar plate containing 100 μg/ml ampicillin. The plate was incubated at 37°C overnight. To confirm correct DNA sequences, single colonies were sent for direct colony sequencing (Genewiz). Correct clones were then purified and transformed into *E. coli* BL21 (DE3) competent cells using a similar transformation procedure as described above. Glycerol stocks were made and stored at −80°C until ready to use.

### Heterologous Protein Expression

The BL21 (DE3) glycerol stock of each construct was used to inoculate a 5 ml culture of 1X LB media with 100 μg/ml ampicillin in a 15 ml sterile test tube. The culture was grown at 37°C overnight with shaking in a GeneMate Mini shaker at 200 rpm. The next day, 2 ml of the overnight culture was added into a baffled 1 L flask containing 200 ml of 1X Terrific Broth with 100 μg/ml ampicillin. Cells were grown at 37°C with shaking in a Thermo Scientific MaxQ 4450 shaker at 200 rpm until the OD_630nm_ reached 0.6–0.8. The flask was then placed on ice until cool and 0.5 mM isopropyl β-d-thiogalactopyranoside (IPTG) was added to induce protein expression. The culture was then shaken on a Thermo Scientific MaxA 2000 shaker at 150 rpm at RT for 16 h. Cells were harvested by centrifugation at 4500 rpm in a Thermo Scientific ST 8R centrifuge at 4°C for 30 min. The supernatant was discarded and the pellet was resuspended in 30 ml of lysis buffer (0.01 M Tris HCl pH 8.3, 0.5 M NaCl, 5 mM imidazole, 5% glycerol, 5 mM beta-mercaptoethanol (BME)). This mixture was kept on ice and sonicated using a Branson 450 Digital Sonifier. The program was set for a total of 5 min of sonication with 10 s on and 10 s off at 10% amplitude. Afterwards, the crude extracts were stored at −80°C.

### Protein Purification

Proteins were purified using nickel affinity chromatography via the following procedure. Crude extracts were thawed and centrifuged at 15,000 rpm in a Sorvall SS-34 rotor at 4°C for 50 min. The ÄKTA™ Start FPLC was equilibrated with 10 CVs of buffer A (10 mM Tris HCl pH 8.3, 500 mM NaCl, 5 mM BME) and the supernatant was loaded onto a 1 ml HisTrap FF column using the sample pump. The column was washed with 5 CVs of buffer A with 25 mM imidazole to remove non-specific binding proteins. The tagged protein was eluted with 5 CVs of buffer B (10 mM Tris HCl pH 8.3, 500 mM NaCl, 5 mM BME, 500 mM imidazole) and then desalted using a PD-10 column (GE Healthcare) and buffer A. Next, Tobacco Etch Virus (TEV) protease (purified as described previously (Czub et al., 2018)) was used to cleave the polyhistidine tag from the protein construct. A total of 1 ml of sample containing buffer A, the tagged protein and TEV (mass ratio was 20:1 for tagged protein:TEV) was added into the top cup of a Slide-a-Lyzer mini dialysis tube (Thermo Fisher Scientific) and 14 ml of cleavage buffer (50 mM Tris HCl pH 8.3, 300 mM NaCl, 0.5 mM EDTA, 5% glycerol, 2.5 mM DTT) was added to the bottom of the dialysis tube. This tube was placed on an oscillator at 37°C for 2 h and the buffer in the bottom of the tube was exchanged and placed at 4°C overnight. The protein was centrifuged at 4500 rpm at 4°C for 10 min to remove precipitate. The supernatant was combined with buffer A to a total volume of 3 ml to dilute the DTT and then loaded onto a 1 ml HisTrap FF column using a 3 ml injection loop. The cleaved protein was collected as two different peaks: one was the eluate after 5 CVs of buffer A was flowed over the column, and the second was during a 0–30% gradient of buffer B over 10 CVs. Proteins were desalted into buffer A without BME and concentrated using Amicon 10kD MWCO centrifugation tubes (Millipore). Protein from the second peak was used for all kinetic assays. Absorbances of proteins at A_280nm_ were measured using a Thermo Scientific Nanodrop Spectrophotometer and protein concentrations were calculated using the monomer molecular weight of 20,675 Da, and an extinction coefficient of 20,400 M^-1^cm^-1^. The purity of the proteins and cleavage of the tag were confirmed by SDS-PAGE and proteins were stored at −80°C. All constructs were soluble and were purified to near homogeneity (Supplementary Figure S2).

### Steady-State Enzyme Kinetics

All constructs were screened for enzymatic activity toward spermine using a previously described colorimetric assay (Kuhn et al., 2013) with the following modifications. Each 50 µL reaction contained 70 mM bicine pH 8.0, 20 mM NaCl, 0.5 mM AcCoA, 3 mM spm, and 10 µL of enzyme (0.1 μg/μL) was used to initiate the reaction in a 96 well clear polystyrene microplate. The reaction proceeded at 37°C for 5 min and 50 µL of solution (6 M guanidine HCl and 0.1 M Tris HCl pH 8.0) was added to terminate the reaction. Next, 200 µL of Ellman’s reagent (0.2 mM 5,5-dithio-bis-(2-nitrobenzoic acid) (DNTB), 0.1 M Tris HCl pH 8.0, 1 mM EDTA) was added and incubated for 10 min at RT. The amount of CoA produced from each reaction was determined spectrophotometrically by measuring the absorbance of the samples at 415 nm and standard solutions of cysteine. Substrate saturation curves were performed on all constructs that exhibited activity during the screening assay, by varying spm concentrations (0–3 mM) and holding AcCoA concentration constant (0.5 mM). Protein concentrations used for substrate saturation curves were: WT (0.07 µM), R1C1 (1.2 µM), R1C2 (0.14 µM), R1C3 (3.6 µM), R3C1 (2.5 µM), R3C2 (17 µM), R3C4 (0.1 µM), R3C5 (0.1 µM), and R3C6 (0.2 µM) During screening, R3C3 and R1C4 had very low to no activity above baseline, so we did not perform a substrate saturation curve for these constructs. Data were collected for at least two biological replicates for each construct, except R1C3 and were fitted to the Hill equation using Prism 9.0.0. The S_0.5_ is the concentration of substrate at half the maximal velocity.

### Crystallization

Proteins were expressed and purified using procedures previously described in (Tsimbalyuk et al., 2020). All crystallization trials were performed using the hanging drop vapor diffusion method in 48-well plates (Hampton Research). Each coverslip contained 1.5 µL of protein and 1.5 µL of reservoir solution suspended over 300 µL of reservoir solution. A detailed list of final crystallization conditions used are outlined in Supplementary Table S2. Crystals were cryoprotected in reservoir solution with 20% glycerol before being flash-cooled at 100 K in liquid nitrogen.

### Data Collection, Data Processing and Structure Determination

X-ray diffraction data were collected using the MX1 crystallography beamline (Eiger 2 9M detector) at the Australian Synchrotron (McPhillips et al., 2002; Cowieson et al., 2015)*.* The data were indexed and integrated in iMosflm (Battye et al., 2011), merged and scaled using AIMLESS (Evans and Murshudov, 2013). Phaser (McCoy, 2007) was used for phase determination and molecular replacement, and the *V. cholerae* SpeG protein structure (PDB ID: 4mi4) was used as a search model. The model was rebuilt in Coot (Emsley et al., 2010), and refined using Refmac5 (Murshudov et al., 2011; Kovalevskiy et al., 2018) and Phenix (Adams et al., 2010; Afonine et al., 2012). Structures were validated using Phenix. R1C2, R1C3, R3C1, R3C4, R3C5 and R3C6 structures were deposited into the Protein Data Bank with accession codes 7kwh, 7kwj, 7kwq, 7kwx, 7kx2, and 7kx3, respectively.

### Homology Modeling

Two homology models for each construct were built using the Swiss-Model server (https://swissmodel.expasy.org/interactive#structure) (Waterhouse et al., 2018). One model was based on the apo- WT VcSpeG structure PDB ID: 4jjx and the other model was based on the spm-bound VcSpeG structure PDB ID: 4mi4. The quality of each model was evaluated using QMEAN (Qualitative Model Energy Analysis) and GMQE (Global Model Quality Estimation) scores (Supplementary Table S3).

## Results

### Defining Key Regions of VcSpeG for Investigation

The VcSpeG and hSSAT1 proteins both acetylate spm and spd, but they have different quaternary structures, i.e. VcSpeG is a dodecamer (Filippova et al., 2015a), whereas hSSAT1 is a dimer (Zhu et al., 2006). The hSSAT1 protein is a domain-swapped dimer and the active site contains residues from both protomers (Zhu et al., 2006), but the dimer within the VcSpeG dodecamer does not exhibit swapping and the active site is contained within each monomer (Filippova et al., 2015a) (Figure 1). Unlike VcSpeG, the hSSAT1 protein does not exhibit an allosteric polyamine binding site.

**FIGURE 3:**
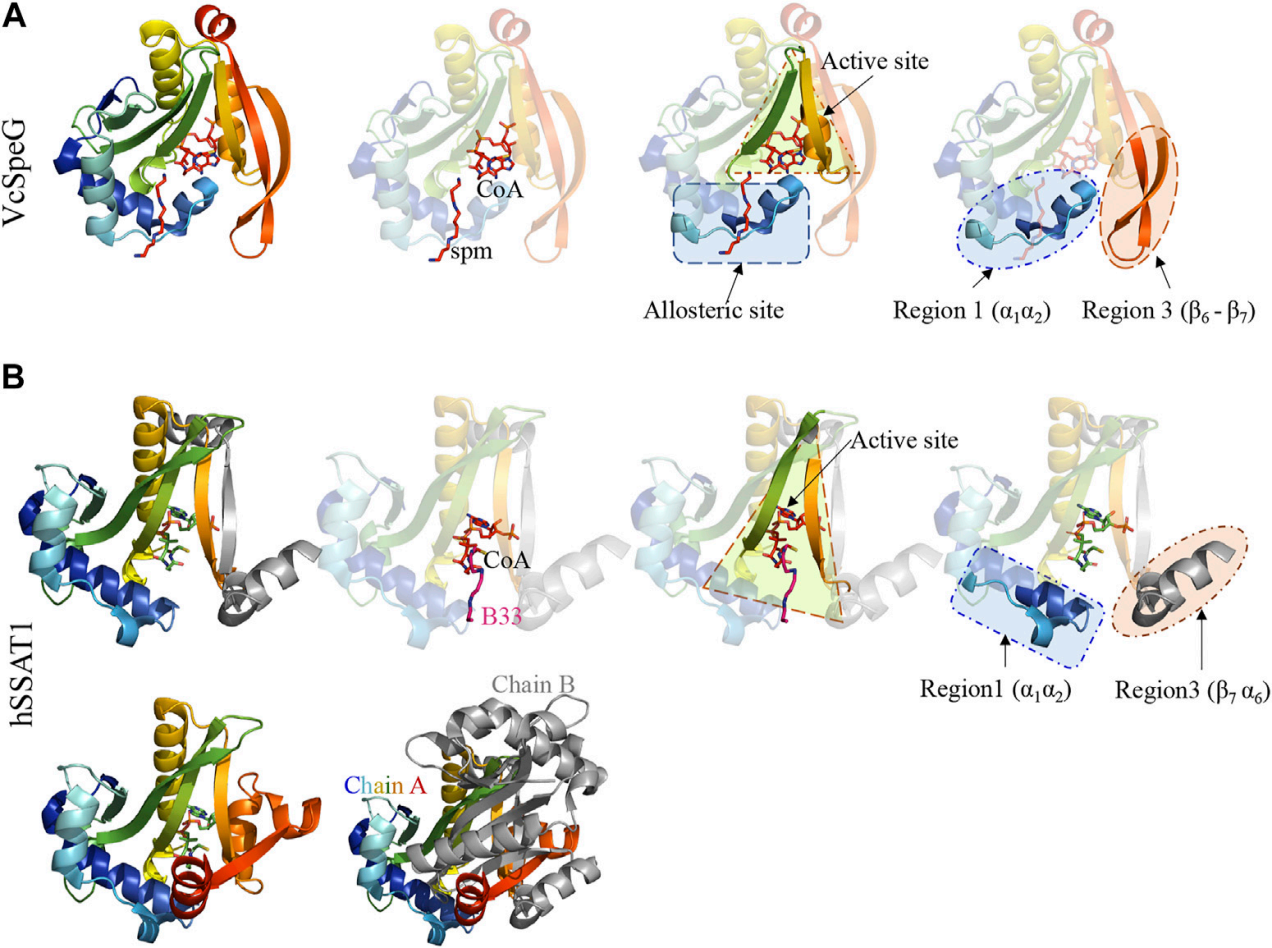
Comparison of VcSpeG and hSSAT1 structures. Each structure is colored in rainbow from N-terminus to C-terminus from blue to red. **A**. A monomer of the VcSpeG PDB ID: 4r87 structure is shown as a ribbon diagram and ligands CoA and spm are shown as sticks. The allosteric site, active site, and Regions 1 and 3 of the protein structure are shown. Spm is bound to the allosteric site, whereas CoA is bound to the active site. **B.** A single monomer (chain A) of the hSSAT1 protein structure PDB ID: 2b4b is shown in rainbow colors and the second monomer (chain B) of the dimer is in gray. The second monomer (chain B) except the secondary structure that is swapped into the first monomer has been removed for clarity to show the location of the active site. The active site of this protein has CoA and the inhibitor N^1^ N^11^-bis-(ethyl)-norspermine shown as sticks. The regions that correspond to Regions 1 and 3 of the VcSpeG protein structure are shown.

This allosteric site in VcSpeG is located between adjacent monomers of the hexamer and interacts with spm using residues N22, E33, E34, Y36, E37, E41 of one monomer and H49, I50, D52, and E55 of an adjacent monomer; there is a loop in the allosteric site (residues 23–38) that transitions to a helix upon binding spm (Filippova et al., 2015a). While the hSSAT1 and VcSpeG enzymes have low sequence identity (21%), they exhibit significant structural similarity, with the exception of three main regions: Region 1 is the VcSpeG allosteric loop (α1- α2). Region two is an insertion between β2 and β3 in the hSSAT1 protein that is not present in the VcSpeG enzyme, and Region 3 is the C-terminal portion of the protein (β6-β7 in VcSpeG and β7-α6 in hSSAT1) that interacts with the allosteric loop of VcSpeG (Figures 2, 3). Therefore, we investigated the contribution of the allosteric loop (Region 1) and portions of the β6-β7 residues (Region 3) of VcSpeG on its kinetic activity. This was accomplished by substituting these portions of the VcSpeG protein with comparable residues of the hSSAT1 protein (i.e. constructing chimeric proteins); we also created selected point mutations of the VcSpeG enzyme (Figure 2). Our primary focus was on Regions 1 and 3 of the VcSpeG protein (Figure 2) since we hypothesized the combination of these two regions would be important for forming the active conformation of VcSpeG.

### Strategy for Generating VcSpeG Constructs

To investigate these previously unstudied regions of the VcSpeG protein, we used a structure-guided approach. Our strategy was to first select and test a subset of key point mutants and chimeric constructs. We created six chimeras (R1C1-R1C4, R3C1-R3C2), one construct with a deletion of a specific region of the protein (R3C3), and three single point mutants (R3C4-R3C6) (Figure 2). These constructs were named based on the region where the residue(s) are located in the VcSpeG protein and were numbered sequentially, e.g. R3C2 corresponds to a change in Region 3 and is the second construct made to query the importance of this region of the VcSpeG protein. A cartoon version of the protein sequences is shown in Figure 2 and full protein sequences of these ten constructs can be found in Supplementary Figure S1 and Table S1.

#### Chimeric Constructs in Region 1 of VcSpeG: The Importance of Specific Regions of the Allosteric Loop (R1C1-R1C4)

The allosteric loop of VcSpeG is sixteen residues in length. To determine which residues of the hSSAT1 protein should be substituted for VcSpeG residues of this region (Region 1), we overlaid the spm-bound VcSpeG structure (PDB ID: 4r87) and the hSSAT1 structure (PDB ID: 2b4b) and performed a sequence alignment between these two proteins (Figure 2). Residues of the α1-α2 helices of the hSSAT1 protein overlaid with the allosteric loop (Region 1) of the VcSpeG protein (Figure 3). Since this loop was relatively long, we created a series of four constructs to investigate the importance of this region on the activity of VcSpeG. R1C1 replaces all residues of this region with the hSSAT1 protein sequence, R1C2 replaces the first half of the residues, R1C3 replaces the first three quarters of the residues, and R1C4 replaces the latter half of the residues of this region (Figure 2).

#### Chimeric Constructs and Deletion in Region 3 of VcSpeG: The Importance of the Beta-Turn Between **β**6-**β**7 (R3C1-R3C3)

The C-terminal portions of the protein sequences of VcSpeG and hSSAT1 adopt quite different conformations. This portion of the hSSAT1 protein is intercalated in the opposite monomer of the dimer (domain-swapped), whereas the entire sequence of the single polypeptide chain of VcSpeG is contained within a single monomer (no domain-swapping) (Figure 1). In the linear protein sequence, Region 3 of these proteins corresponds to β6-β7 of VcSpeG and β7-α6 of hSSAT1 (Figure 3). We created three constructs to investigate the importance of the β6-β7 region of VcSpeG on its activity. The first construct (R3C1) removes the residues that form a portion of β7 and a portion of the beta-turn of the β6-β7 region of the protein and replaces them with the corresponding residues from hSSAT1 in its linear sequence (Figure 2). Since the N152 residue of VcSpeG is replaced with a positively charged lysine from hSSAT1 and it normally forms an H-bond with R25 of Region 1, it is possible this substitution would repel the interaction between these two regions. The second construct (R3C2) substitutes a larger portion of the β6-β7 region of VcSpeG with the hSSAT1 sequence (Figure 2). This construct would enable us to determine if the protein can withstand large alterations in this region and still be active, and/or whether these types of alterations are sufficient to cause the VcSpeG protein to adopt domain-swapping like the hSSAT1 protein. The final construct (R3C3) is a deletion of the entire portion of the VcSpeG protein sequence that was substituted in the R3C2 construct and has glycine residue insertion (Figure 2).

#### Point Mutations in Region 3 of VcSpeG: The Importance of Residues That Potentially Stabilize Interactions Between Regions 1 and 3 (R3C4-R3C6)

**FIGURE 4:**
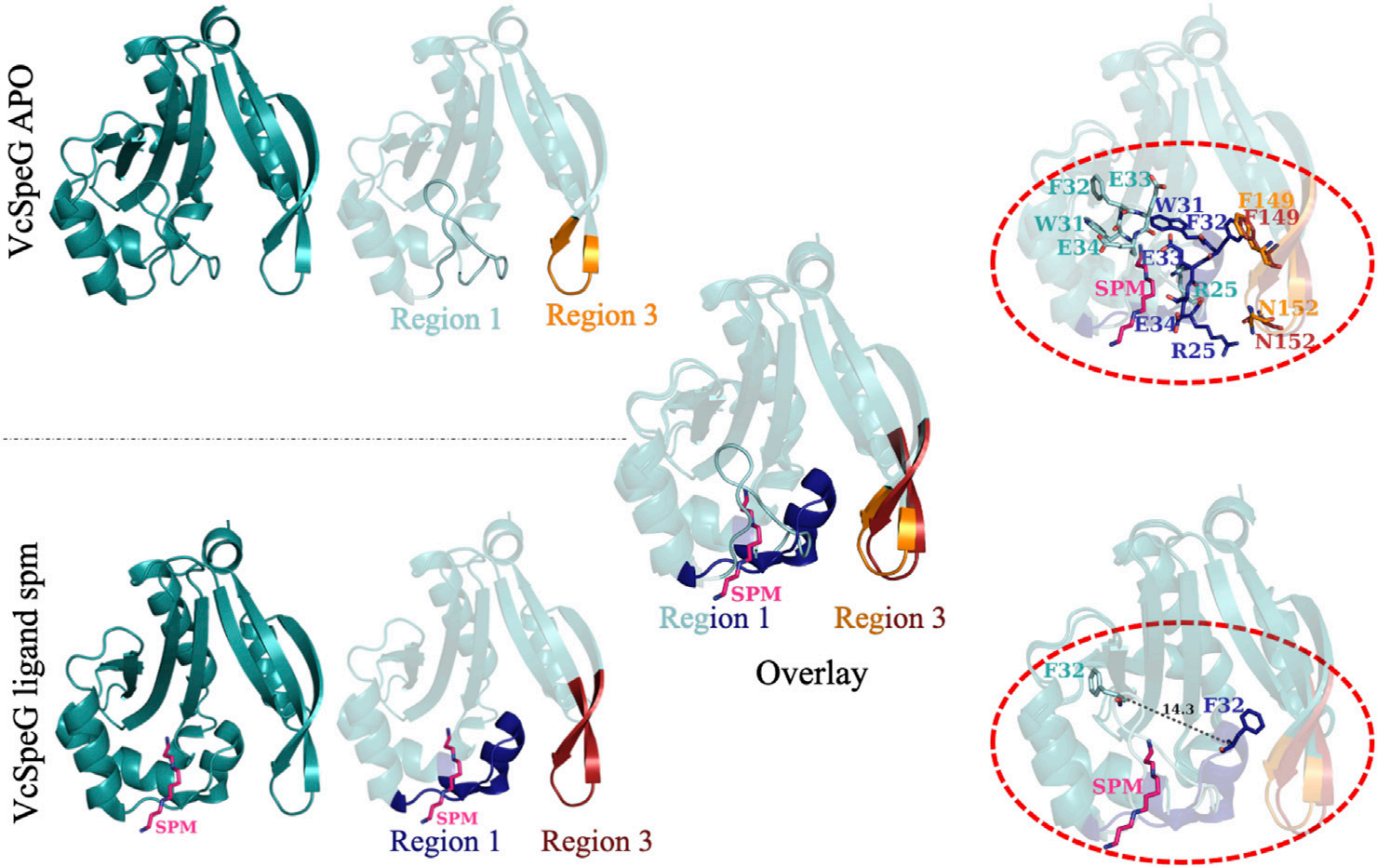
Comparison of structural changes of the allosteric loop of the VcSpeG protein in absence and presence of spermine. The VcSpeG monomer in presence (PDB ID: 4mi4) and absence of spm (PDB ID: 4jjx) is shown in ribbon diagrams. The spm ligand is shown as pink sticks and nitrogens are in dark blue. Region 1 in the apo form is shown in cyan, but the same region in the spm-bound structure is shown in dark blue. Region 3 from the apo structure is in orange and is in red for the spm-bound structure. An overlay of these two structures is shown to indicate movement of Region 1 when spm is present. Residues that alter their conformation significantly upon this transition (R25, W31, F32, E33, E34, F149, and N152) are shown in the top dotted oval in sticks and are colored according to structures and regions from which they are found. The bottom dotted oval shows the distance between the C_α_ of F32 in the apo- and spm-bound structures is 14.3 Å.

When polyamine binds to the allosteric site of VcSpeG, a significant conformational change of the allosteric loop (Region 1; α1-α2) occurs compared to the apo-structure, and Region 1 moves closer to Region 3 (β6-β7) (Figure 4). Indeed, the distance between the alpha carbon of F32 on this allosteric loop in the apo- (PDB ID: 4jjx) and the alpha carbon of F32 on the spm-bound (PDB ID: 4mi4) structures is 14.3 Å (Figure 4). Our analysis of the spm-bound structure of VcSpeG showed these two regions form potentially significant contacts: π-π interactions between F32 and F149 and an H-bond between R25 and N152. To elucidate whether these residues could be important for stabilizing the interaction between these two regions of VcSpeG, we created three point mutants: N152L, F149A, and F149G (R3C4-R3C6; Figure 2). We substituted leucine for asparagine at position 152 to retain the overall structural similarity of the residue but remove the ability to H-bond, and we substituted alanine and glycine for phenylalanine at position 149 to potentially decrease the ability of this residue to form hydrophobic interactions.

### Crystallization Trials and Structure Determination

In order to confirm the structural integrity and assess the conformation of the chimeric and mutant proteins, we attempted to determine the structure of all ten constructs using X-ray crystallography. All ten constructs were solubly expressed in *E. coli* and purified using a two step purification method. Purified proteins were screened for conditions that induce crystallization, and a total of six crystal structures were determined: R1C2 (PDB ID: 7kwh), R1C3 (PDB ID: 7kwj), R3C1(PDB ID: 7kwq), R3C4 (PDB ID: 7kwx), R3C5 (PDB ID: 7kx2), and R3C6 (PDB ID: 7kx3) at the resolution 2.9Å, 2.58Å, 2.3Å, 2.42Å, 2.6Å, and 2.67Å, respectively. Detailed data collection and refinement statistics of the solved structures can be found in the Supplemental Information Supplementary Tables S4, S5. All substituted residues of the VcSpeG protein sequences of these constructs were ordered in the structures, with the exception of R1C3, which had ARYEYME residues of the substitution that were disordered (Figure 2 and Supplementary Figure S3). Despite crystallizing in a range of crystallography space groups (Supplementary Table S4, S5), six structures adopted the apo-conformation of VcSpeG (Figure 4 and Supplementary Figure S3). Since all constructs were soluble and the majority were able to be crystallized, it appears the alterations to the VcSpeG sequence did not cause the protein to improperly fold.

### Generation of Homology Models of Constructs in Presence and Absence of Ligand

**FIGURE 5:**
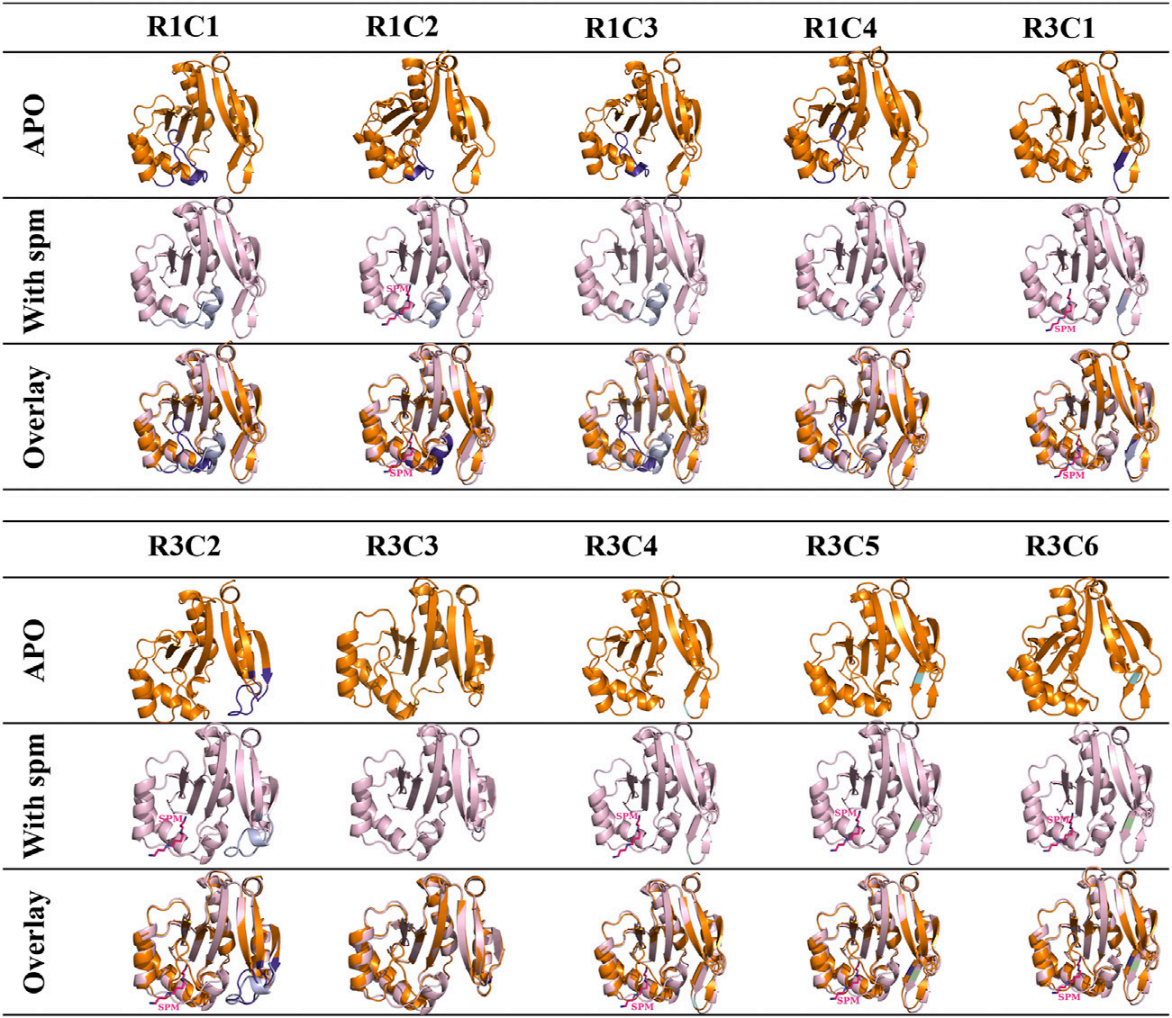
Analysis of apo and spm-bound homology models of 10 VcSpeG constructs. Homology models of all constructs were generated using Swiss model to obtain apo- and spm-bound models since some proteins did not crystallize. Apo-homology models are in orange ribbon diagrams and the WT-apo structure (PDB ID: 4jjx) was used as the template. The spm-bound homology models are in light pink and were based on the WT with spm-bound structure (PDB ID: 4mi4) as the template. Substitutions are shown in purple for the apo models and violet for the spm-bound models. Light green indicates the location of a point mutation and spm is shown as pink sticks. Spm was present in the R1C2, R3C1, R3C2, R3C4, R3C5, and R3C6 models.

Since we did not obtain crystals for all constructs and none had spm bound, we built homology models of each construct in the presence and absence of spm (Figure 5). The homology models of the apo-constructs were generated using the PDB ID: 4jjx WT VcSpeG structure as the template, and the homology models of the spm-bound constructs used the spm-bound PDB ID: 4mi4 WT VcSpeG structure as the template. All models were of good quality as defined by their QMEAN and GMQE scores (Supplementary Table S3). Our rationale for generating homology models of the apo constructs was three-fold: 1) some residues in the crystal structures were disordered and the placement of certain residues could not be determined, 2) the structures of some constructs could not be determined or they did not crystallize, and 3) we wanted to determine if the homology models generated were similar to the crystal structures of constructs as a way to validate the homology model predictions.

Our analysis of the apo-crystal structures and corresponding homology models of the monomer showed good root mean square deviations (rmsd) between the two (R1C2 rmsd 1.45 Å, R3C1 rmsd 1.17 Å, R3C4 rmsd 1.01 Å, R3C5 rmsd 1.25 Å, and R3C7 rmsd 1.20 Å), with the exception of R1C3 which had an rmsd of 4.33 Å. Minor differences in residue positions were observed in the overlaid R1C2 (K23-E29) and R3C1 (R149, L150, F151, K152) crystal structures and models. However, the R1C3 crystal structure had a disordered region (K23-Q34) which contributed to the higher rmsd value for the overlaid apo-structure and homology model (Supplementary Figure S3). We also generated homology models for the remaining constructs that had not been crystallized in the apo-form and found their rmsd values ranged from 0.36–1.56 Å (Supplementary Table S6).

Next, we generated homology models of the constructs based on the spm-bound VcSpeG crystal structure. The R1C2, R3C1, R3C2, R3C4, R3C5, R3C6 homology models retained spm bound to the allosteric site in the models, whereas R1C1, R1C3, R1C4, R3C3 did not. Similar to the apo crystal structures and homology models, the rmsd values for the homology models based on the spm-bound WT crystal structure were quite good. They ranged from 0.86–1.06 Å for all constructs, with the exception of R3C2, which had an rmsd of 1.84 Å (Supplementary Table S6). This discrepancy was based on an alteration in secondary structure predicted in the homology model compared to the WT crystal structure (described below). Based on these results, and the fact that the WT VcSpeG structure in the presence of spm has been determined previously, we felt confident the homology models could be used as a reasonably accurate tool for rationalizing our kinetic results and predicting potential structural effects of altering these regions of the VcSpeG protein in absence of crystal structures. Of course, crystal structures, binding studies, and/or molecular dynamics simulations of these constructs in the presence of spm are needed to confirm or disprove our predictions based on the homology models.

### Kinetic Characterization of all VcSpeG Constructs

All proteins, including the WT enzyme, were screened for their enzymatic activity toward spm. With the exception of R3C3, all constructs exhibited measurable activity above baseline under saturating conditions. Therefore, substrate saturation curves were constructed and fits were performed to obtain estimates of the kinetic parameters of the constructs.

#### VcSpeG F149A, F149G and N152L Point Mutations Exhibit Relatively Minor Effects on Enzyme Kinetic Activity

**FIGURE 6:**
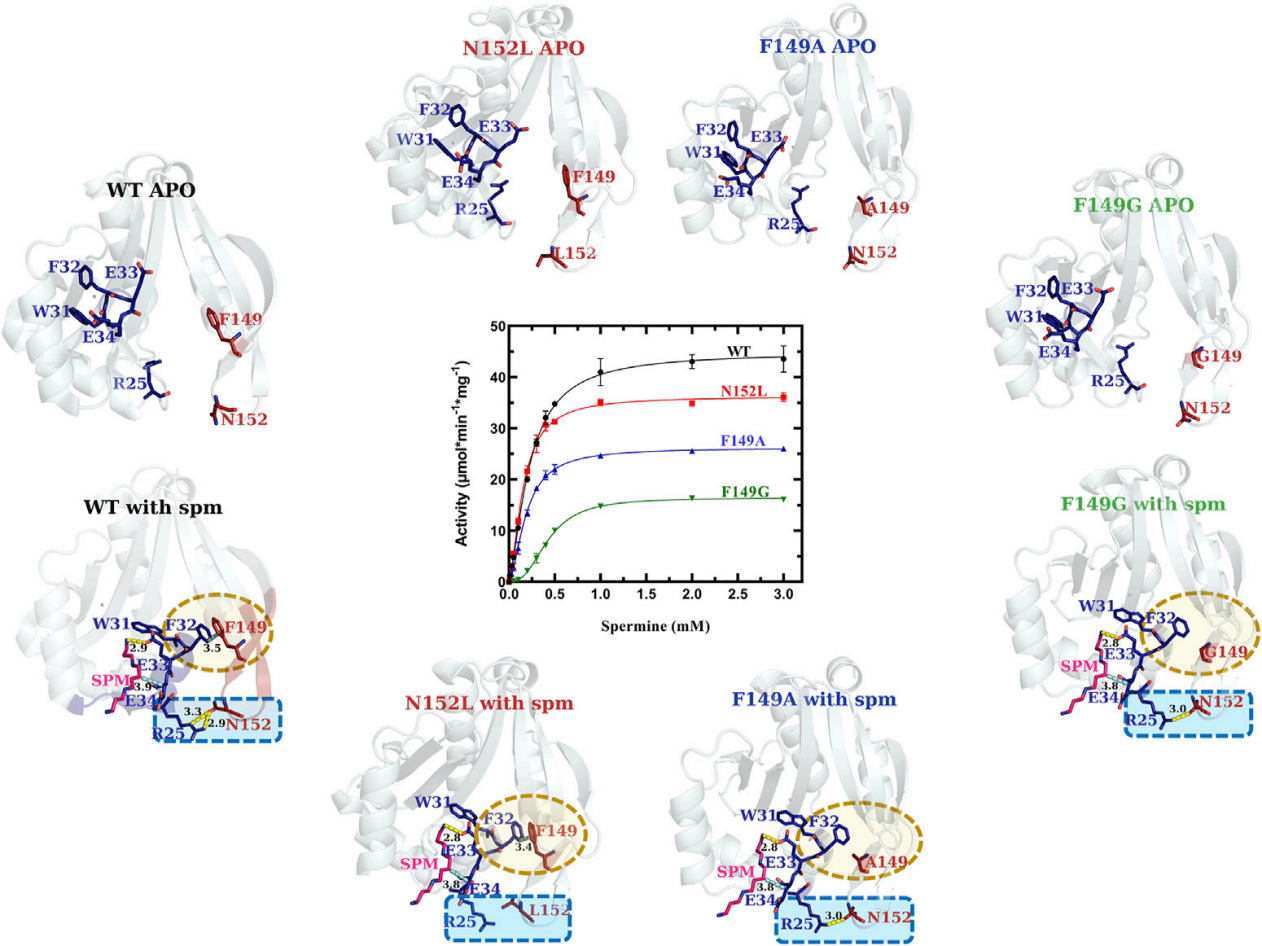
Comparison of kinetic activity and structures/models of Region 3 point-mutants (R3C4-R3C6). Crystal structures of apo-WT, R3C4 (N152L), R3C5 (F149A), and R3C6 (F149G) are shown along the top of the substrate saturation kinetic plot, whereas homology models of R3C4-R3C6 constructs based on the spm-bound WT crystal structure (PDB ID: 4mi4) are along the bottom. Regions of variability are highlighted in yellow and blue dotted shapes. H-bonds are shown as dashed yellow lines. Specific residues of interest are shown as sticks and labeled.

In the WT VcSpeG structure, two main interactions between Regions 1 and 3 occur: an H-bond between R25 and N152, and π-π interactions between F32 and F149. During our kinetic analyses we found the VcSpeG N152L (R3C4), F149A (R3C5), and F149G (R3C6) mutant enzymes still retained reasonably high activity toward spm (Figure 6 and Table 1). The catalytic efficiency of the N152L mutant increased slightly (1.2-fold), and the F149A and F149G mutants decreased 1.4- and 5.3-fold, respectively compared to WT. The relatively minor changes in catalytic efficiency were driven predominately by alterations to k_cat_.

**TABLE 1:** Kinetic parameters of WT VcSpeG and ten constructs.

		S_0.5_ (mM)	k_cat_ (s^−1^)	k_cat_/S_0.5_ (M^−1^ s^−1^)	*n*
	WT	0.222 ± 0.005	15.5	6.97 × 10^4^	1.47 ± 0.05
Allosteric loop chimera	R1C1	2.53 ± 0.71	1.2	4.58 × 10^2^	0.91 ± 0.06
	R1C2	0.364 ± 0.008	9.3	2.54 × 10^4^	2.17 ± 0.11
	R1C3	0.244 ± 0.020	0.3	1.33 × 10^3^	1.01 ± 0.06
	R1C4	ND*			
β6-β7 region chimera	R3C1	0.459 ± 0.016	0.6	1.29 × 10^3^	1.97 ± 0.12
	R3C2	0.098 ± 0.008	0.1	5.24 × 10^2^	1.23 ± 0.10
	R3C3	ND*			
β6-β7 region point mutants	R3C4	0.153 ± 0.004	12.5	8.17 × 10^4^	1.59 ± 0.06
	R3C5	0.187 ± 0.003	9	4.83 × 10^4^	1.68 ± 0.04
	R3C6	0.430 ± 0.006	5.7	1.32 × 10^4^	2.60 ± 0.09

*None-detected.

Our analysis of the spm-bound homology models for the R3C4-R3C6 constructs showed all three retained spm in the allosteric site (Figure 6). Based on these models, a substitution of leucine for asparagine at position 152 (R3C4) caused the H-bond between side chains of R25 and N152 to be disrupted, while the π-π interactions between F32 and F149 side chains were retained. On the other hand, when we replaced phenylalanine with alanine (R3C5) and with glycine (R3C6) at position 149, the π-π interactions between F32 and F149 were removed and the H-bond between R25 and N152 side chains remained. While this H-bond is still possible in the R3C6 homology model, it may ultimately be disrupted due to the greater flexibility of a glycine substitution at position 149. In contrast, an alanine substitution at position 149 still has hydrophobic character and may be sufficient for the H-bond between R25 and N152 to be retained as shown in the homology model. Overall, these results provide a basis for understanding why the mutations at these positions did not completely disrupt the enzyme’s ability to catalyze the reaction.

#### The VcSpeG **β**6-**β**7 Region Is Critical for Enzyme Kinetic Activity

**FIGURE 7:**
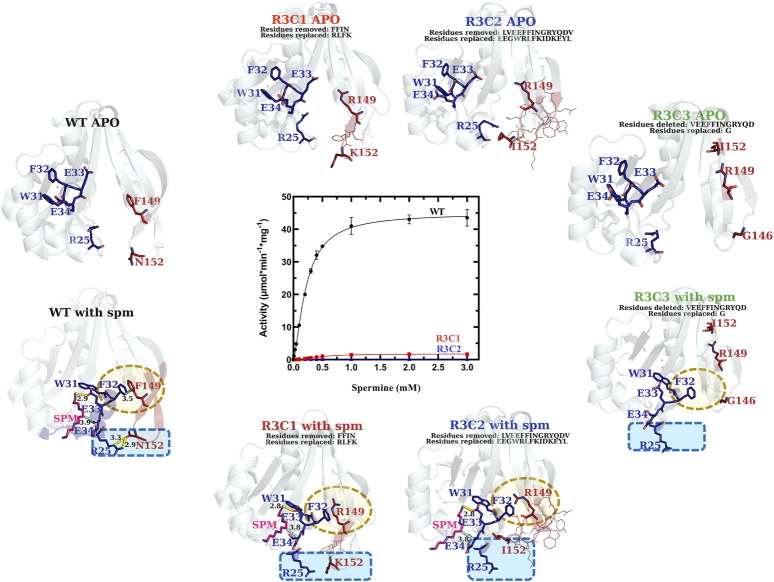
Comparison of kinetic activity and structures/models of Region 3 chimera and deletion constructs (R3C1-R3C3). The apo-crystal structure of R3C1 and homology models of R3C2 and R3C3 are shown along the top of the substrate saturation kinetic plot, whereas homology models of R3C1-R3C3 constructs based on the WT spm-bound crystal structure (PDB ID: 4mi4) are along the bottom. Regions of variability are highlighted in yellow and blue dotted shapes. H-bonds are shown as dashed yellow lines. Specific residues of interest are shown as sticks and labeled and lines of less interest are shown as lines and are not labeled.

In contrast to the single point mutations we tested, exchanging larger portions of Region 3 (R3C1-R3C3 constructs) caused dramatic effects on enzyme activity (Figure 7 and Table 1). The replacement of the beta-turn (four amino acids; FFIN (F149-N152)) of Region 3 of the VcSpeG protein (R3C1) caused the activity to decrease 26-fold compared to WT. Additionally, replacement of a more significant portion (13 amino acids) of the β6-β7 region of the protein (R3C2), caused the activity to decrease 155-fold compared to WT. Comparatively, the catalytic efficiency of these two constructs (R3C1 and R3C2) decreased 54-fold and 133-fold, respectively compared to WT, indicating k_cat_ exhibited a larger influence on catalytic efficiency than S_0.5_. The R3C3 construct was inactive.

We analyzed the homology models that were generated based on the spm-bound VcSpeG structure for these constructs and saw both R3C1 and R3C2 had spm bound in the allosteric site, but R3C3 did not (Figure 7). As expected, the homology model of the R3C3 construct showed the core of the protein structure remained unchanged, but 14 amino acids of Region 3 of the protein were not present. The R3C1 model showed the secondary structures of this region generally adopted the same conformation as WT. When we examined the residues of this construct compared to the WT protein more closely, the homology models showed substitutions of F149 to arginine and F150 to leucine affect their interactions with Region 1. For example, the models showed R149 formed an H-bond with the adjacent E148 residue, which disrupted the π-π interactions originally held by F32 and F149. Additionally, when F150 was replaced by leucine in the model, the T-shaped π-π interaction originally formed between F150 and Y155 in the WT structure was disrupted. Substitution of N152 to lysine also removed the H-bond typically seen between R25 and N152 in the WT structure (Supplementary Figure S4).

In the R3C2 homology model, the substituted region of this construct adopts a more helical character for residues EGWRL (146–150), which is in contrast to the longer beta-strands in this region of the WT protein (Supplementary Figure S5). This indicates larger substitutions within this region of the protein could potentially cause changes to secondary structure. The conformation of the secondary structure that contains this substitution appears to be stabilized by H-bonds between the side chain of E155 and backbone amines of E126, N127, and D153, and an H-bond between the backbone oxygen of Y156 and backbone amine of V125. Note, E155 and Y156 correspond to tyrosine and glutamine, respectively in the WT structure and are located on opposite sides of the beta-strand. They do not form these H-bonds seen in the R3C2 construct homology model (Supplementary Figure S5).

#### Specific Portions of the VcSpeG Allosteric Loop Are Critical for Enzyme Kinetic Activity

**FIGURE 8:**
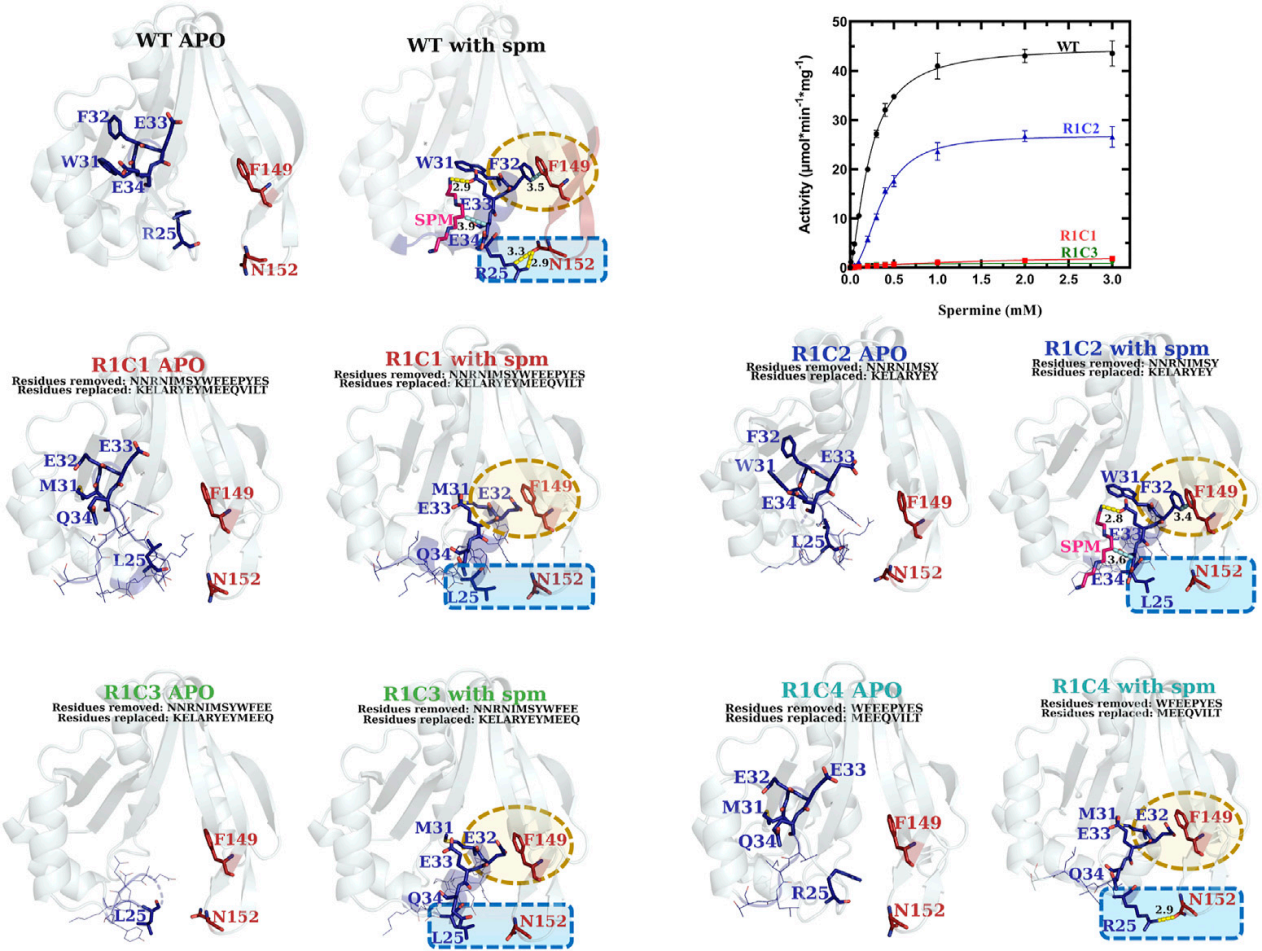
Comparison of kinetic activity and structures/models of Region 1 chimeric constructs (R1C1-R1C4). The apo-crystal structures of R1C2 and R1C3 and homology models of R1C1 and R1C4 are shown adjacent to homology models of R1C1-R1C4 constructs based on the WT spm-bound crystal structure (PDB ID: 4mi4) and are labeled in different colors for clarity. Regions of variability are highlighted in yellow and blue dotted shapes. H-bonds are shown as dashed yellow lines. Specific residues of interest are shown as sticks and labeled and lines of less interest are shown as lines and are not labeled. A plot of the substrate saturation curves for these constructs and WT proteins is also shown.

Our examination of systematic allosteric loop substitutions (Region 1) on VcSpeG kinetic activity showed: 1) substituting large regions of this loop caused dramatic decreases in activity and 2) using this approach enabled us to narrow down the region of the loop that had the most significant effect on activity (Figure 8 and Table 1). Substituting the entire 16 residue allosteric loop with residues from the hSSAT1 protein (R1C1) decreased activity 13-fold compared to WT. On the other hand, only substituting the first eight residues of this loop (R1C2) caused a minimal (1.7-fold) decrease in activity compared to WT. Alternatively, when the first three quarters of the loop or latter half of the loop residues were substituted (R1C3 and R1C4, respectively), the enzyme activity decreased by 52-fold for R1C3 and no activity was detected for the R1C4 construct. A more drastic decrease in catalytic efficiency was observed for the R1C1 construct (two orders of magnitude compared to WT) compared to the R1C3 construct (one order of magnitude compared to WT). This difference is due to the fact that the R1C1 enzyme did not reach complete saturation under the described reaction conditions and exhibited a one order of magnitude lower apparent affinity for spm compared to the R1C3 and WT enzymes.

Based on the kinetic results, it became clear that the region of the allosteric loop that was critical for activity was between residues 31–34 (WFEE). Therefore, we examined the homology models for the Region 1 constructs to determine whether we could glean information from the structures that might explain why this region was important (Figure 8). In the WT structure, this portion of the allosteric loop forms several important interactions, including an NH-arene interaction between Q86 and W31 (Supplementary Figure S6), π-π interactions between F32 of Region 1 and F149 of Region 3, an H-bond between the terminal amine of spm and the sidechain of E33 in the allosteric site, and a hydrophobic interaction between E34 and the methylenes of spm in the allosteric site. The R1C1, R1C3, and R1C4 constructs all have MEEQ in place of WFEE, which would disrupt these interactions and may explain the reduced activity of these constructs. On the other hand, the R1C2 construct retained these residues and may explain why this construct retained activity.

## Discussion

### Chimeric Constructs as a Tool to Study SSAT Enzymes

Our strategy for using chimeric constructs to probe the importance of different regions of a protein of interest is not new. In fact, chimeric proteins are used in all sorts of applications, including bioengineering (Adlakha et al., 2011), vaccine development (Nuccitelli et al., 2011), cancer treatments (Qiu et al., 2008), and as drugs for type 2 diabetes (Findeisen et al., 2019). Chimeric genes arise naturally during evolution and enable formation of different protein functions and the diversification of protein structures (Rogers and Hartl, 2012). Here, we have used chimera to provide a broad overview of potential regions of the VcSpeG protein that are important for its activity and structure. We specifically investigated the contribution of the VcSpeG allosteric loop and adjacent β6-β7 region on its activity. Our study was primarily centered on enzyme kinetics and homology models of the constructs based on the spm-bound VcSpeG structure. It is possible that some constructs are unable to bind spm, and/or they do not adopt the conformation of the allosteric loop observed in the models. Regardless, these models show the types of interactions that may be important for an active state of VcSpeG and form the basis for future queries across different polyamine acetyltransferases and the broader GNAT family.

### Two Previously Unexplored Regions of VcSpeG Are Critical for Activity

**FIGURE 9:**
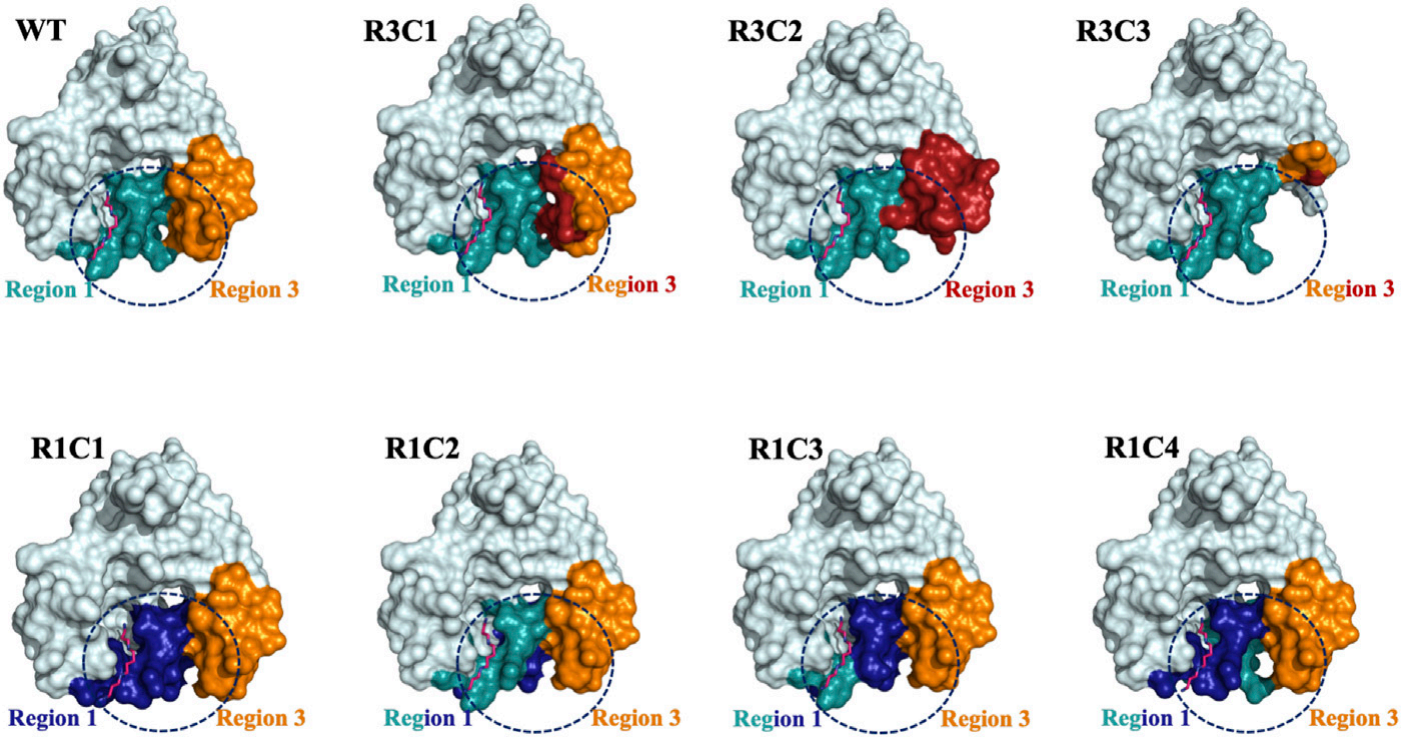
Surface representations of selected Region 1 and 3 constructs. The surface effects of residue substitutions for R1C1-R1C4 and R3C1-R3C3 constructs compared to the WT protein are shown. These diagrams are based on the homology models of the spm-bound WT VcSpeG structure (PDB ID: 4mi4). Region 1 (allosteric loop) is shown in teal and Region 3 is in orange in all models and the WT structure. Residues exchanged from hSSAT1 in Region 1 are shown in dark blue and residues exchanged in Region 3 are in red. The dashed circle emphasizes the surface alterations to these regions of all constructs compared to the same area in the WT VcSpeG protein.

Our results show both the allosteric loop (Region 1) and the β6-β7 residues (Region 3) of the VcSpeG protein are important for VcSpeG activity. Furthermore, substituting these regions with hSSAT1 residues, with the exception of R1C2, caused major decreases in enzyme activity. The investigation of alterations to Region 1 of the VcSpeG protein (R1C1-R1C4) showed we could replace residues in the first half of the allosteric loop (R1C2) and retain moderate activity, but exchanging residues in either the second half of the loop (R1C4), the first three quarters of the loop (R1C3) or the entire loop (R1C1) caused the enzyme to be inactive. Therefore, residues within the WFEE portion of the allosteric loop are critical for activity, but we do not know whether all residues or only a subset of these four residues are required for activity toward spm for the VcSpeG enzyme. These four residues are conserved across all structurally characterized SpeG enzymes, and Sugiyama *et al.* previously showed point mutants of the *E. coli* SpeG enzyme in this comparable region were critical for activity toward spd (Sugiyama et al., 2016). However, we currently do not know whether the VcSpeG allosteric site must bind polyamine for turnover to occur and which residues in the allosteric site might transmit this signal when spm or spd is bound. Regardless, it is clear from our homology models that the conformation of the allosteric loop is important for forming adequate interactions with Region 3. Furthermore, a tightening between these two regions of the VcSpeG protein occurs in the WT structure that appears to be disrupted by substitutions to either Region 1 or Region 3 in our chimeric constructs (Figure 9).

The single point mutants we tested in Region 3 (F149, N152; R3C4-R3C6) were insufficient to totally inhibit enzyme activity. However, when we examined the chimera with a four-residue substitution (R3C1), including both of the F149 and N152 residues exchanged with positively charged residues, we saw a much more substantial decrease in activity. While it is possible a single amino acid substitution to this region may be enough to alter kinetic activity, it appears the contribution of multiple residues, largely driven by F149, is responsible for decreased activity. In the homology models, we saw these substitutions altered H-bonds and π-π interactions with residues in Region 1, which may be important for forming a more closed conformation of these two regions of the protein (Figure 9). H-bonds are important for protein structure and function, including protein folding, protein-ligand recognition, and enzymatic activity (Jeffrey and Saenger, 1991; Myers and Pace, 1996; Nick Pace et al., 2014). Additionally, when residues are buried in non-polar environments, π-π, cation-π, and amino-π interactions have been recognized for their vital roles in protein structure and stability (Burley and Petsko, 1986; Gallivan and Dougherty, 1999; Dougherty, 2007; Dalkas et al., 2014; Vernon et al., 2018; Thakuria et al., 2019). Therefore, the combination of π-π and H-bond interactions between Regions 1 and 3 appear to be quite important for VcSpeG enzyme activity and are driven by the proper conformation of the allosteric loop.

The replacement or deletion of the entire Region 3 of the VcSpeG protein completely inhibits the enzyme’s ability to turnover. Our homology models showed large substitutions in Region 3 caused changes to the secondary structure and surfaces of this region (Figure 9). Thus, the reduction in activity for these constructs is most likely due to removing key interactions with Region 1. Moreover, the R3C2 construct model shows exchanging these hSSAT1 residues opens up the space between Regions 1 and 3, and creates new interactions in Region 3 not observed in the VcSpeG WT structure or other construct models (Figure 9). In the greater context of GNAT evolution, these results indicate that relatively small changes to this region of GNAT proteins could alter the structure. However, it is possible that substitutions to Regions 1 and/or 3 may be necessary to create specific types of interfaces within or between monomers of the protein.

**FIGURE 10:**
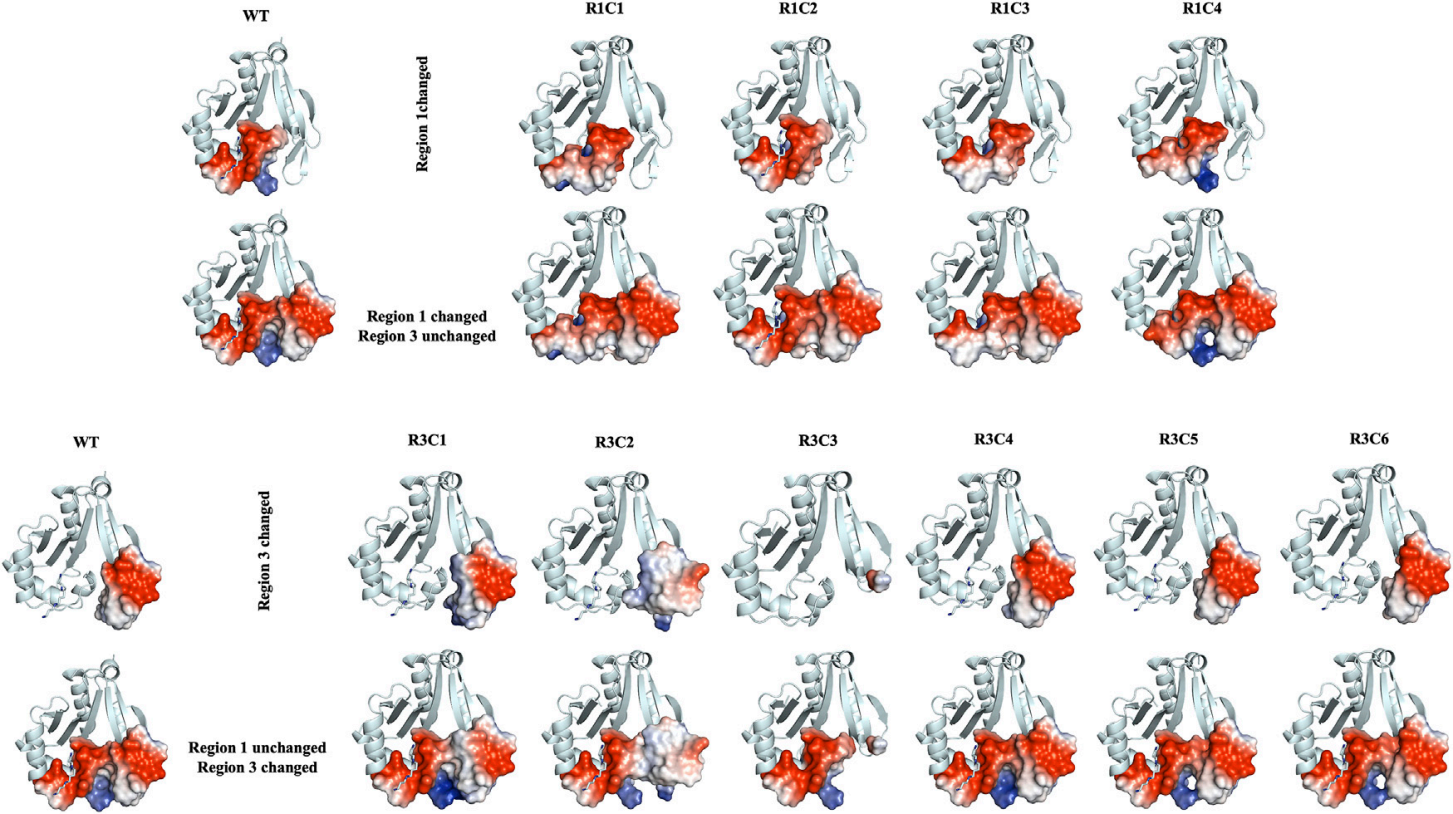
Electrostatic surface representations for Regions 1 and 3 constructs compared to WT VcSpeG. Ribbon diagrams of homology models for each construct are shown and are based on the VcSpeG WT PDB ID: 4mi4 crystal structure with spm bound in the allosteric site. Electrostatic surfaces were calculated in Pymol for Regions 1 and 3 of each construct, where blue represents positive charge, red is negative charge and white is neutral. The upper panel of the figure shows constructs that were modified in Region 1, with Region 3 remaining unchanged, and the bottom panel shows constructs modified in Region 3, with Region 1 remining unchanged.

Further qualitative examination of the electrostatic surfaces of Regions 1 and 3 in the VcSpeG WT and construct models may provide an explanation as to why specific alterations to these two regions of the protein significantly affect enzyme activity (Figure 10). In constructs where the allosteric loop (Region 1) was altered, we observed two key changes compared to WT: 1) the acidity of the allosteric site where polyamine binds was significantly decreased for R1C1, R1C3 and R1C4 constructs and 2) the interface between Regions 1 and 3 became more neutral except for the R1C4 construct. Since the R1C2 construct still maintained significant activity, it appears retention of acidity in the allosteric site may enable polyamine binding and subsequent catalysis, especially when significant van der Waals and complementary electrostatic interactions between Regions 1 and 3 were maintained. This was also observed in constructs where the β6-β7 residues (Region 3) were altered. If the basicity of Region 3 increased (R3C1, R3C2) or the region was completely removed (R3C3), the enzyme activity was obliterated or drastically decreased. In cases where only a single residue in this region was altered (R3C4-R3C6), the disruption to the activity was far less. Thus, it is likely that both the acidity of the allosteric site and complementary interfacial residues between Regions 1 and 3 must be maintained for productive catalysis.

### Rationale for Effects of Allosteric Loop on VcSpeG Activity

When polyamine is bound to the VcSpeG allosteric site, the allosteric loop adopts a different conformation and forms an alpha helix. This conformational change opens up the acceptor site within the active site, creating a pocket for the polyamine acceptor substrate to bind. Therefore, substitutions to residues of the allosteric loop may affect VcSpeG activity in a variety of ways. One possibility is the allosteric loop loses or lessens its ability to bind polyamine at the allosteric site since some residues on this loop protrude into this site. These substitutions could: 1) potentially change the overall surface charge of the predominantly negatively charged binding site, or 2) create steric effects that could prevent or alter polyamine binding, affecting the allosteric signal. A second possibility is the allosteric loop may still adopt the helical conformation without requiring polyamine binding, but lessen turnover due to absence of an allosteric signal. Alternatively, these changes could cause the allosteric loop to block the active site, either through inability to adopt the proper loop conformation or inability to bind polyamine, thus inhibiting enzyme activity. Ultimately, a specific conformation of the allosteric loop appears to be required for interaction with Region 3 of the VcSpeG protein for catalysis to proceed efficiently. Molecular dynamics simulations may help to clarify these effects and should be explored in the future. Clearly, much remains to be learned about this enzyme system.

## Data Availability

The datasets presented in this study can be found in online repositories. The names of the repository/repositories and accession number(s) can be found below: http://www.wwpdb.org/, 7kwh http://www.wwpdb.org/, 7kwj http://www.wwpdb.org/, 7kwq http://www.wwpdb.org/, 7kwx http://www.wwpdb.org/, 7kx2 http://www.wwpdb.org/, 7kx3.
