# Supplementary material for: The Vibrio cholerae SpeG Spermidine/Spermine N-Acetyltransferase Allosteric Loop and β6-β7 Structural Elements Are Critical for Kinetic Activity
Source: Front Mol Biosci. 2021 Apr 13;8:645768. doi: 10.3389/fmolb.2021.645768 (PMC8076852; doi:10.3389/fmolb.2021.645768)
Supplement: Supplementary file 1 [file datasheet1.docx]

**Supplementary Material**

**Supplementary Figures and Tables**

| **Table S1. Primers used to generate VcSpeG constructs.** F and R refer to the forward and reverse primers. The uppercase letters in the primer indicate the site of substitution. |
| --- |
| \| **Constructs** \| **Name (F/R)** \| **Primers** \| **Tm** \| \| --- \| --- \| --- \| --- \| \| R1C1 \| p066_R1C1_F  p067_R1C1_R \| atggaagaacaggtgattctgaccTTTGATGAACTCGAAGAG  atattcatagcgcgccagttctttATTGAGATTGTGAATAAAGC \| 55°C  55°C \| \| R1C2 \| p068_R1C2_F  p069_R1C2_R \| cgctatgaatatTGGTTTGAAGAGCCTTATG  cgccagttctttATTGAGATTGTGAATAAAGC \| 59°C  55°C \| \| R1C3 \| p070_R1C3_F  p071_R1C3_R \| gaatatatggaagaacagCCTTATGAATCCTTTGATGAAC  atagcgcgccagttctttATTGAGATTGTGAATAAAGC \| 58°C  58°C \| \| R1C4 \| p072_R1C4_F  p073_R1C4_R \| gtgattctgaccTTTGATGAACTCGAAGAG  ctgttcttccatGTAGGACATAATGTTGCG \| 55°C  58°C \| \| R3C1 \| p074_R3C1_F  p075_R3C1_R \| tttaaaGGTCGTTACCAAGATGTTAAG  caggcgTTCCTCGACTAAGTGGCC \| 61°C  64°C \| \| R3C2 \| p076_R3C2_F  p077_R3C2_R \| aaaattgataaagaatatctgAAGAGGATGTATATACTGCAAAG  aaacaggcgccagccttcttcGTGGCCTTCCTCTACAAATC \| 60°C  63°C \| \| R3C3 \| p078_R3C3_F  p079_R3C3_R \| gtgAAGAGGATGTATATACTGCAAAG  gccTAAGTGGCCTTCCTCTAC \| 60°C  61°C \| \| R3C4 \| p080_R3C4_F  p081_R3C4_R \| ATTTTTCATCctgGGTCGTTACCAAGATGTTAAGAG  TCCTCGACTAAGTGGCCT \| 60°C  65°C \| \| R3C5 \| p082_R3C5_F  p083_R3C5_R \| AGTCGAGGAAgcgTTCATCAACGGTCG  AAGTGGCCTTCCTCTACA \| 58°C  63°C \| \| R3C6 \| p084_R3C6_F  p085_R3C6_R \| AGTCGAGGAAggcTTCATCAACGGTCG  AAGTGGCCTTCCTCTACA \| 59°C  63°C \| |

**Table S2. Crystallization conditions for VcSpeG constructs**

| **Construct** | **Reservoir solution** | **Stock concentration** |
| --- | --- | --- |
| R1C2 | 0.1M MES pH6.5, 10% PEG5000 MME, and 12%propanol | 10 mg/ml |
| R1C3 | 2M sodium formate, 0.1M sodium acetate pH5.0 | 10 mg/ml |
| R3C1 | Deionized water | 2 mg/ml |
| R3C4 | 10% ethanol | 10 mg/ml |
| R3C5 | 0.2M potassium chloride pH 7.0, 20% PEG3350 | 10 mg/ml |
| R3C6 | 0.1M Sodium malonate, 30% PEG 3350 | 10 mg/ml |

| **Table S3. GMQE and QMEAN scores of 10 construct homology models.** A QMEAN score between -4 and 0 and a GMQE score between 0-1 are indicative of an accurate and reliable model, respectively. |
| --- |
| \|  \| WT template  (PDB ID: 4jjx) \| \| Spm-bound template  (PDB ID: 4mi4) \| \| \| --- \| --- \| --- \| --- \| --- \| \| Construct \| GMQE \| QMEAN \| GMQE \| QMEAN \| \| R1C1 \| 0.87 \| -1.24 \| 0.87 \| -1.18 \| \| R1C2 \| 0.89 \| -1.16 \| 0.98 \| -0.70 \| \| R1C3 \| 0.89 \| -0.76 \| 0.88 \| -0.61 \| \| R1C4 \| 0.88 \| -1.47 \| 0.89 \| -0.92 \| \| R3C1 \| 0.88 \| -1.36 \| 0.98 \| -0.76 \| \| R3C2 \| 0.75 \| -1.90 \| 0.98 \| -1.45 \| \| R3C3 \| 0.87 \| -2.04 \| 0.85 \| -0.65 \| \| R3C4 \| 0.89 \| -1.56 \| 0.98 \| -0.82 \| \| R3C5 \| 0.89 \| -1.24 \| 0.98 \| -0.79 \| \| R3C6 \| 0.90 \| -1.44 \| 0.98 \| -0.92 \| |
|  |

**Table S4. Data collection and refinement statistics for R1C2 (7kwh) and R1C3 (7kwj)**

|  | **7kwh** | **7kwj** |
| --- | --- | --- |
| **Resolution range** | 39.68– 2.90(2.97 - 2.90) | 29.97 – 2.70(2.58 – 2.58) |
| **Space group** | P 21 21 21 | I 2 2 2 |
| **Unit cell** | 105.45 156.53 188.73 | 73.90 134.97 138.78 |
| **Total reflections** | 483379(30524) | 37165 (3617) |
| **Unique reflections** | 69880(4434) | 1878 (202) |
| **Multiplicity** | 6.9(6.9) | 4.0(3.8) |
| **Completeness (%)** | 99.9(99.9) | 98.3(93.5) |
| **Mean I/sigma(I)** | 7.9(2.0) | 13.6(2.3) |
| **Wilson B-factor** | 66.24 | 54.84 |
| **R-merge** | 0.182(1.621) | 0.056(0.404) |
| **R-meas** | 0.197(1.748) | 0.064(0.466) |
| **R-pim** | 0.074(0.644) | 0.030(0.227) |
| **CC1/2** | 0.996(0.509) | 0.998(0.919) |
| **Reflections in refinement** | 69805 (6893) | 21697 (2024) |
| **Reflections for R-free** | 3566 (357) | 1158 (123) |
| **R-work** | 0.2179 (0.3123) | 0.2349 (0.2647) |
| **R-free** | 0.2547 (0.3674) | 0.2782 (0.3298) |
| **Non-hydrogen atoms** | 16962 | 4066 |
| **macromolecules** | 16962 | 4066 |
| **ligands** | - | - |
| **Protein residues** | 1999 | 489 |
| **RMS(bonds)** | 0.001 | 0.001 |
| **RMS(angles)** | 0.37 | 0.37 |
| **Ramachandran**  **favored**  **allowed (%)** | 97.11  2.89 | 96.86  3.14 |
| **outliers (%)** | 0.00 | 0.00 |
| **Rotomer outliers (%)** | 0.06 | 0.23 |
| **Clashscore** | 1.74 | 3.75 |
| **Average B-factor** | 68.25 | 61.54 |
| **macromolecules** | 68.25 | 61.54 |
| **ligands** | 79.56 | - |

*Statistics for the highest-resolution shell are shown in parentheses.

**Table S5. Data collection and refinement statistics for R3C1 (7kwq), R3C4 (7kwx), R3C5 (7kx2), and R3C6 (7kx3)**

|  | **7kwq** | **7kwx** |
| --- | --- | --- |
| **Resolution range** | 49.18 - 2.38(2.30 - 2.30) | 48.66 - 2.51(2.42 - 2.42) |
| **Space group** | I 2 2 2 | I 2 2 2 |
| **Unit cell** | 74.89 137.90 140.35 | 73.02 135.94 139.40 |
| **Total reflections** | 150471(15036) | 235035(23707) |
| **Unique reflections** | 32692(3160) | 26869(2762) |
| **Multiplicity** | 4.6(4.8) | 8.7(8.6) |
| **Completeness (%)** | 100.0(100.0) | 100.0(99.7) |
| **Mean I/sigma(I)** | 16.4(2.4) | 16.1(2.4) |
| **Wilson B-factor** | 49.91 | 46.32 |
| **R-merge** | 0.056(0.404) | 0.092(0.892) |
| **R-meas** | 0.064(0.466) | 0.098(0.949) |
| **R-pim** | 0.030(0.227) | 0.033(0.321) |
| **CC1/2** | 0.998(0.919) | 0.998(0.778) |
| **Reflections in refinement** | 21697 (2024) | 26859 (2627) |
| **Reflections for R-free** | 1158 (123) | 1374 (132) |
| **R-work** | 0.2153 (0.3062) | 0.2241 (0.3145) |
| **R-free** | 0.2441 (0.3477) | 0.2733 (0.3810) |
| **Non-hydrogen atoms** | 4375 | 4307 |
| **macromolecules** | 4286 | 4307 |
| **solvents** | 89 | - |
| **Protein residues** | 506 | 510 |
| **RMS(bonds)** | 0.002 | 0.002 |
| **RMS(angles)** | 0.41 | 0.40 |
| **Ramachandran**  **favored**  **allowed (%)** | 98.00  2.00 | 99.21  0.79 |
| **outliers (%)** | 0.00 | 0.00 |
| **Rotomer outliers (%)** | 0.00 | 0.00 |
| **Clashscore** | 0.59 | 1.88 |
| **Average B-factor** | 62.33 | 52.16 |
| **macromolecules** | 62.51 | 52.16 |
| **Solvents** | 53.76 | - |
|  |  |  |
|  | **7kx2** | **7kx3** |
| **Resolution range** | 48.68 (2.60-2.60) | 48.72 - 2.80(2.67 – 2.67) |
| **Space group** | I 2 2 2 | I 2 2 2 |
| **Unit cell** | 73.44 135.94 139.52 | 72.04 136.39 139.23 |
| **Total reflections** | 281833(34945) | 253730(34983) |
| **Unique reflections** | 21877(2623) | 19885(2605) |
| **Multiplicity** | 12.9(13.3) | 12.8(13.4) |
| **Completeness (%)** | 100.0(100.0) | 100.0(100.0) |
| **Mean I/sigma(I)** | 16.7(2.1) | 9.4(2.0) |
| **Wilson B-factor** | 78.48 | 69.71 |
| **R-merge** | 0.091(1.839) | 0.183(2.080) |
| **R-meas** | 0.098(1.988) | 0.199(2.247) |
| **R-pim** | 0.038(0.753) | 0.076(0.846) |
| **CC1/2** | 0.998(0.484) | 0.997(0.384) |
| **Reflections in refinement** | 21876(2120) | 19876 (1931) |
| **Reflections for R-free** | 1166 (128) | 963 (96) |
| **R-work** | 0.2501(0.3733) | 0.2214 (0.3992) |
| **R-free** | 0.2066(0.4218) | 0.2656 (0.3902) |
| **Non-hydrogen atoms** | 4243 | 4275 |
| **macromolecules** | 4238 | 4275 |
| **solvents** | 5 | - |
| **Protein residues** | 504 | 509 |
| **RMS(bonds)** | 0.001 | 0.002 |
| **RMS(angles)** | 0.36 | 0.29 |
| **Ramachandran**  **favored**  **allowed (%)** | 96.39  3.61 | 96.62  3.38 |
| **outliers (%)** | 0.00 | 0.00 |
| **Rotomer outliers (%)** | 0.00 | 0.00 |
| **Clashscore** | 1.56 | 1.67 |
| **Average B-factor** | 85.90 | 72.67 |
| **macromolecules** | 85.92 | 72.67 |
| **Solvents** | 67.62 | - |
|  |  |  |

*Statistics for the highest-resolution shell are shown in parentheses.

**Table S6. Root mean square deviation (RMSD) values for homology models.** The RMSD values were calculated based on the alignment of the homology model to the corresponding WT or spm-bound crystal structures (PDB IDs 4jjx and 4mi4, respectively).

| Construct | RMSD (Å)  apo | RMSD (Å)  spm-bound |
| --- | --- | --- |
| R1C1 | 0.39 | 1.06 |
| R1C2 | 0.39 | 1.04 |
| R1C3 | 0.38 | 1.05 |
| R1C4 | 0.36 | 1.05 |
| R3C1 | 1.25 | 1.04 |
| R3C2 | 1.56 | 1.84 |
| R3C3 | 1.24 | 0.86 |
| R3C4 | 0.38 | 1.03 |
| R3C5 | 0.37 | 1.03 |
| R3C6 | 0.37 | 1.03 |

| **A** | | |
| --- | --- | --- |
| Construct name | Protein sequences | Position of residue |
| 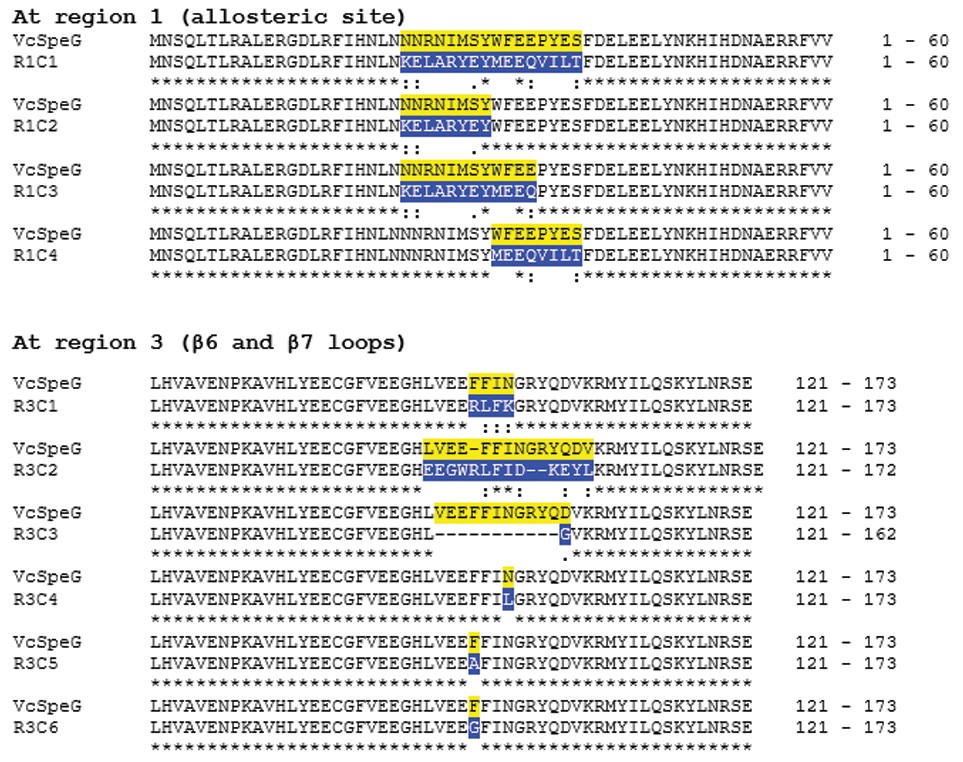 | | |
| 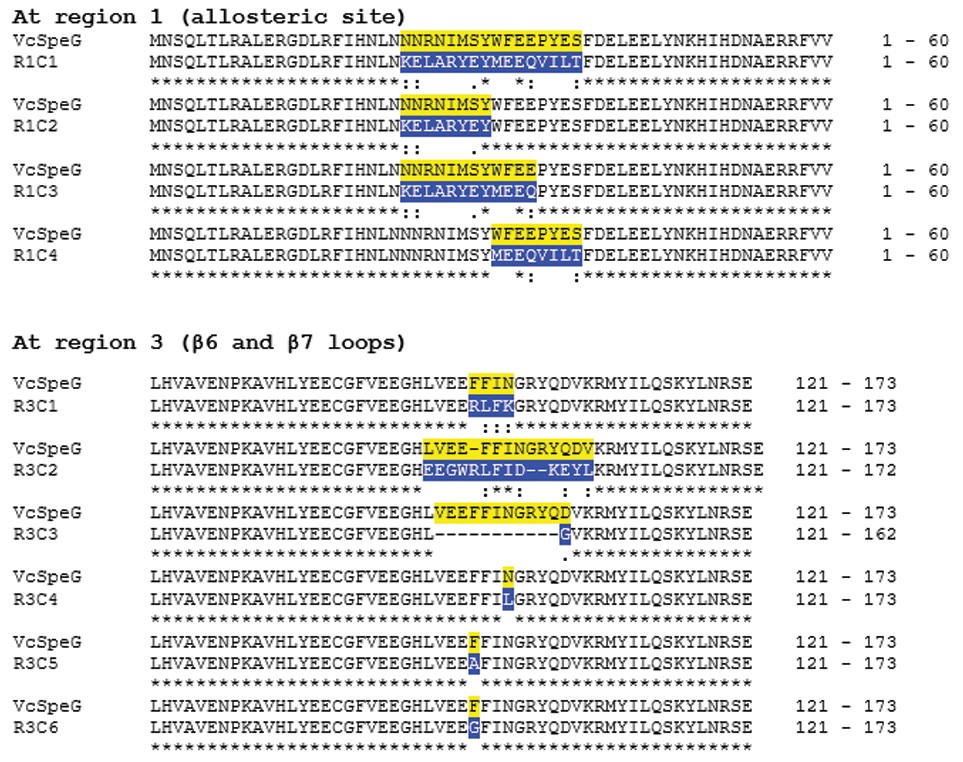 | | |

| **B** |
| --- |
| 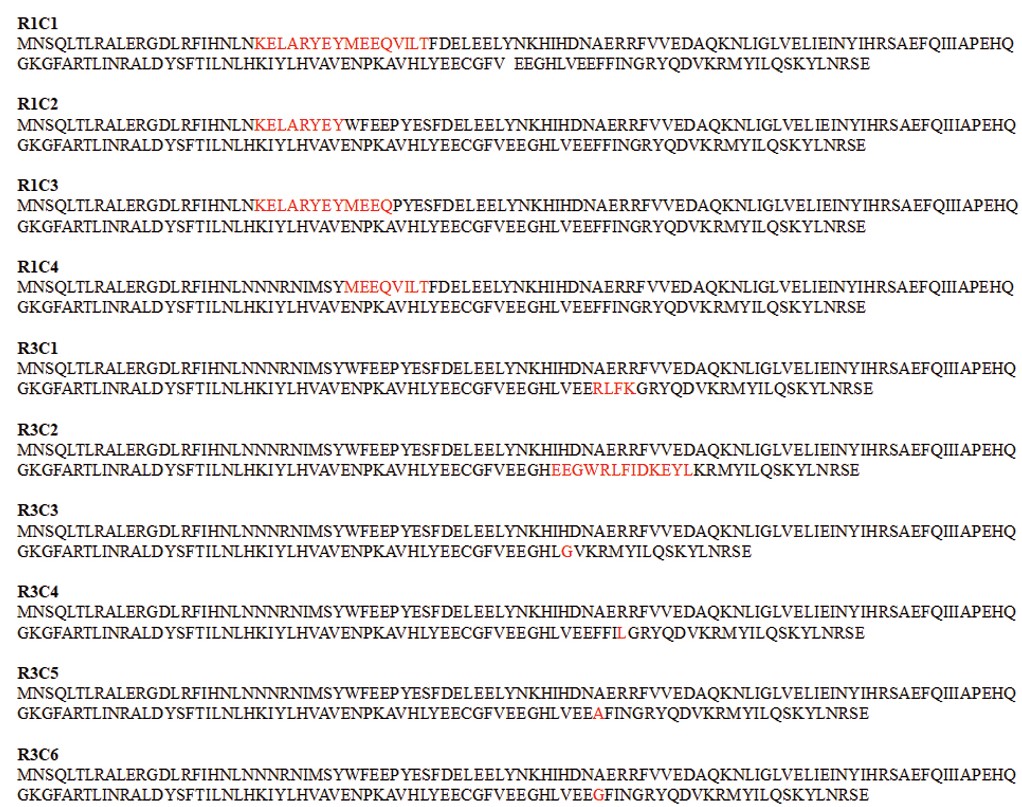 |
|  |

**Figure S1. Protein sequences of 10 constructs. A)** Sequence confirmed protein sequences of WT and constructs. Residues that are different between WT and construct are highlighted in yellow for WT and blue for constructs. **B)** Full protein sequence of each construct. Residues in red correspond to those from hSSAT1, site-directed mutagenesis, or deletion of a specific region.

| 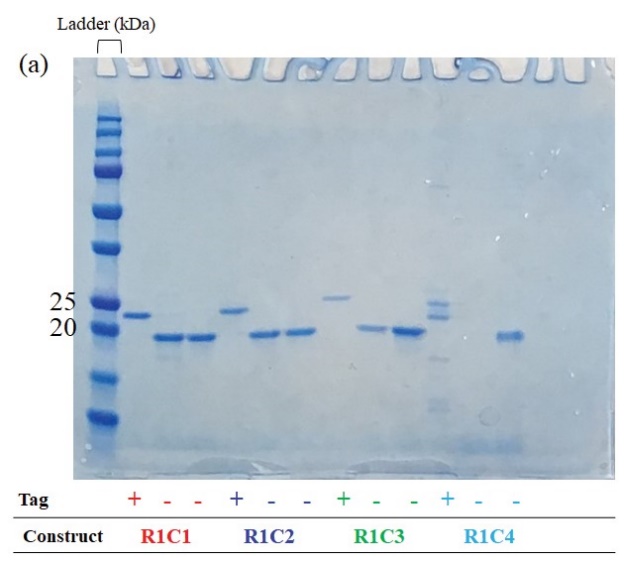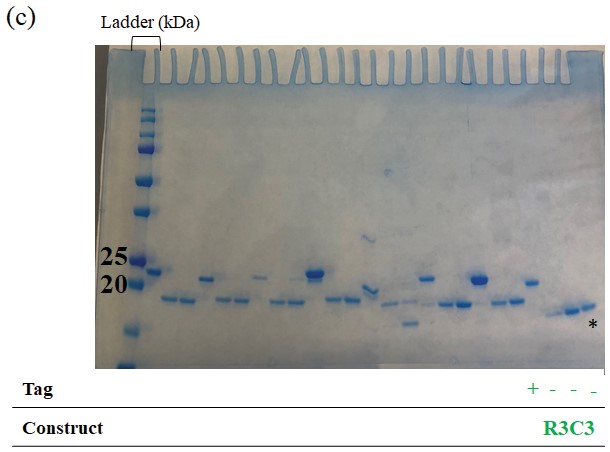 | 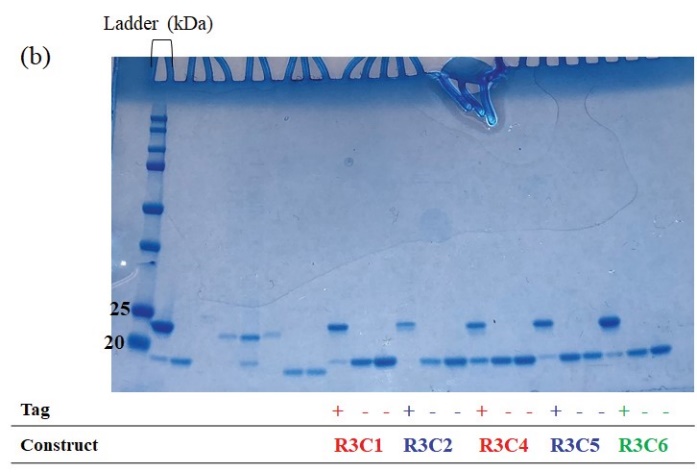 |
| --- | --- |
| **Figure S2. SDS PAGE showing purity of protein constructs.** Each construct was run on an SDS-PAGE under reducing conditions. Samples of each construct in the presence of polyhistidine tag (Tag: +) and absence of polyhistidine tag (Tag: -) after tag cleavage are shown. Each cleaved construct has two samples, the first is the eluate of protein after 5CVs of buffer A flowed over the column and the second is the eluate of the protein during a 0-30% gradient of buffer B over 10 CVs. **(a)** SDS PAGE of constructs R1C1, R1C2, R1C3, and R1C4. **(b)** SDS PAGE of constructs R3C1, R3C2, R3C3, R3C4, R3C5, and R3C6. **(c)** SDS PAGE of construct R3C3. (*) is the same protein in the second negative sign. | |

| 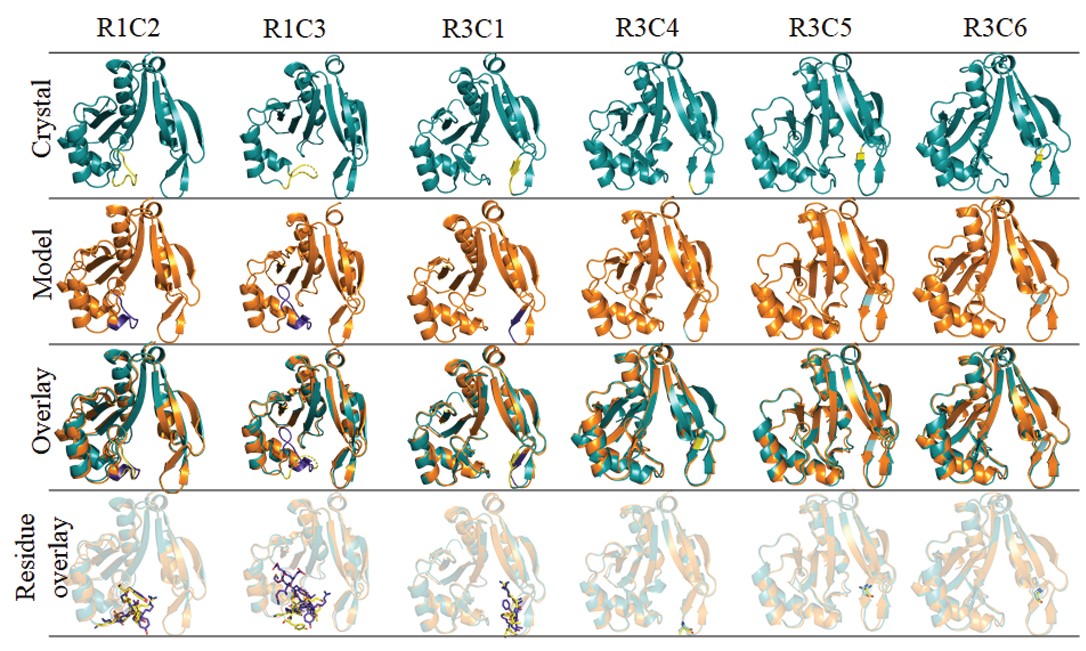 |
| --- |
| **Figure S3. Ribbon diagrams of apo-crystal structures and corresponding homology models.** Crystal structures are shown in teal and homology models are in orange. The regions highlighted in yellow in crystal structures and purple in homology models correspond to residues replaced for each construct. Cyan in models is a point where a residue was mutated. |

| 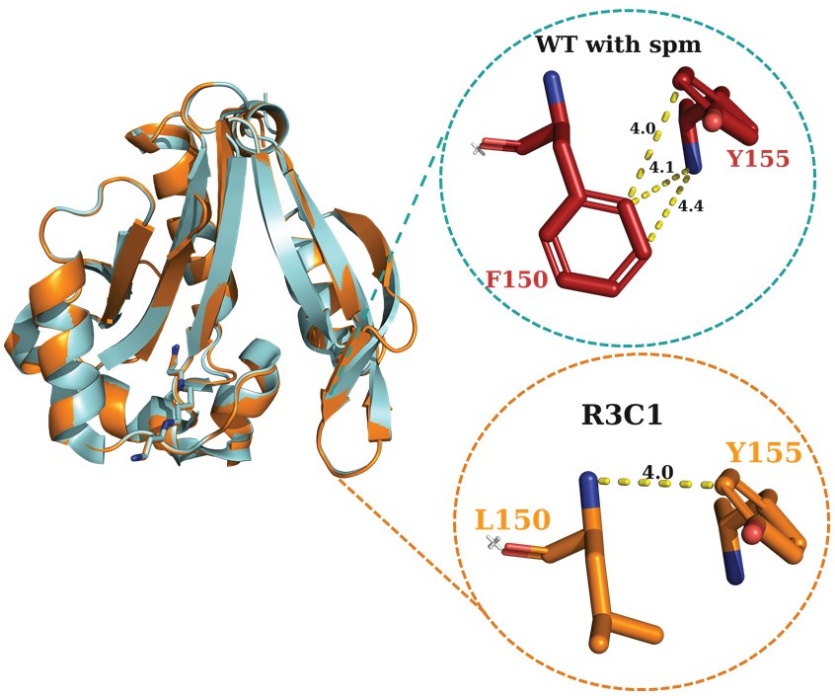 |
| --- |
| **Figure S4. Comparison of amino acid interactions at position 150 of the R3C1 homology spm-bound model and VcSpeG WT spm-bound crystal structure (PBD ID: 4mi4).** Ribbon diagrams show an overlay of the R3C1 spm-bound model (orange) and WT spm-bound structure (cyan). Residues in zoomed view in red sticks belong to the VcSpeG WT spm-bound structure, whereas orange residues are from the R3C1 homology model. A T-shaped amino-π interaction is shown between F150 and Y155 in the WT structure. Yellow dashed lines represent distances between two nearest atoms. |

| 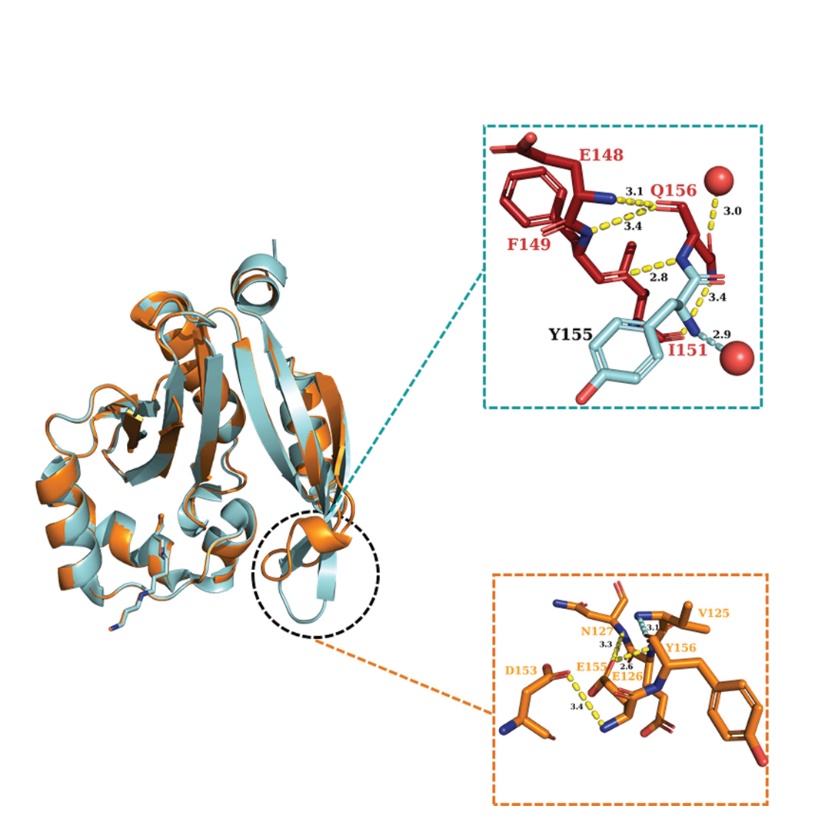 |
| --- |
| **Figure S5**. **Comparison of H-bonds between R3C2 homology spm-bound model and WT spm-bound structure (PBD ID: 4mi4).** Overlay of ribbon diagrams of R3C2 in orange and WT in cyan is shown on the left. Zoomed view shows residues in orange from the R3C2 homology model and residues in red and cyan are from the WT structure. The yellow dashed lines are H-bonds and read spheres are waters. |
| 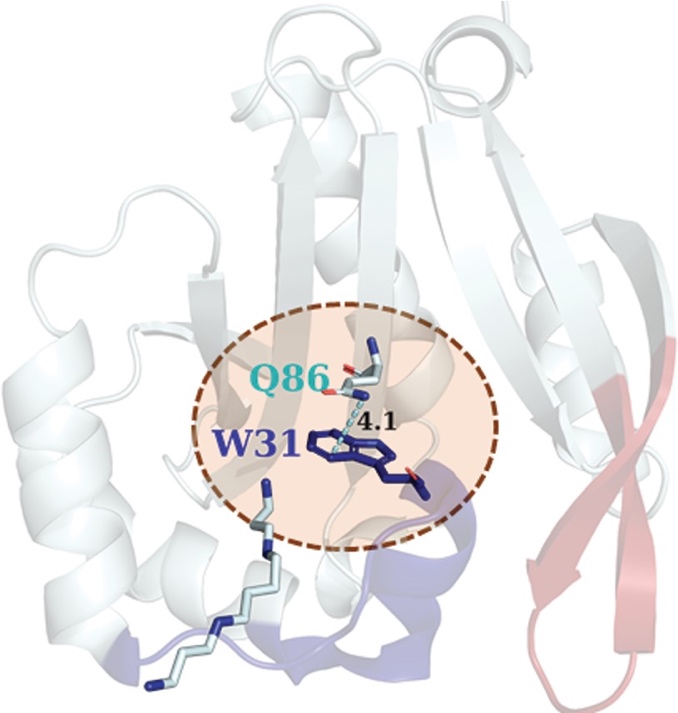 |
| **Figure S6. An NH-arene interaction between Q86 and W31 in the WT VcSpeG spm-bound structure (PBD ID: 4mi4).** VcSpeG monomer ribbon diagram with spm shown as sticks. Blue highlighting indicates Region 1, whereas red highlighting shows Region 3. A cyan dashed line shows the distance (4.1 Å) between two nearest atoms of W31 and Q86. |
